# Bioactive Light-Responsive Au Nanohybrids for Reactive Oxygen Species-Driven Macrophage Reprogramming

**DOI:** 10.1021/acsami.5c10407

**Published:** 2025-09-16

**Authors:** Ting-Yu Cheng, Li-Chan Chang, Li-Xing Yang, Sz-Syuan Wu, Yu-Cheng Chin, Ya-Jyun Chen, Zi-Chun Chia, Wen-Pin Su, Chih-Chia Huang

**Affiliations:** a Department of Photonics, 34912 National Cheng Kung University, Tainan 701, Taiwan; b Institute of Clinical Medicine, College of Medicine, 34912 National Cheng Kung University, Tainan 704, Taiwan; c Departments of Oncology and Internal Medicine, National Cheng Kung University Hospital, College of Medicine, National Cheng Kung University, Tainan 704, Taiwan; d Clinical Medicine Research Center, National Cheng Kung University Hospital, College of Medicine, National Cheng Kung University, Tainan 704, Taiwan; e Center of Applied Nanomedicine, National Cheng Kung University, Tainan 70101, Taiwan; f Department of Medicinal and Applied Chemistry, Kaohsiung Medical University, Kaohsiung 807, Taiwan

**Keywords:** light-responsive nanoparticles, phototherapy, bioactive nanohybrids, macrophage reprogramming, immunotherapy

## Abstract

Optical modulation of immune responses via nanomaterials has emerged as a promising approach in cancer immunotherapy, but challenges in achieving precise activation with minimal phototoxicity persist. In this study, we developed a galactose-functionalized Au–S (2ATP)/polyaniline (PANI)-based glycopolymer nanoparticle (Au/2ATP@PGlyco NP) to enable multivalent galactose-based biostimulation of M2-like macrophages and hot electron/hole-elicited reactive oxygen species (ROS) generation for synergistic macrophage reprogramming. The 2ATP@PANI-based shell not only facilitated light-driven charge transfer to enhance ^1^O_2_ generation but also provided Raman-active properties that enabled single-cell visualization of phenotypic transitions toward the M1 phenotype through the NF-κB and STAT-1-mediated pro-inflammatory signaling. Such light-driven reprogrammed M1-like macrophages with Au/2ATP@PGlyco NP effectively induced apoptosis in MB49 bladder cancer cells through phagocytosis and the release of TNF-α and IL-12, resulting in a potent antitumor effect. This research highlights a new nanobiophotonics platform that holds potential for advancements in macrophage modulation within cancer immunotherapy.

## Graphical abstract

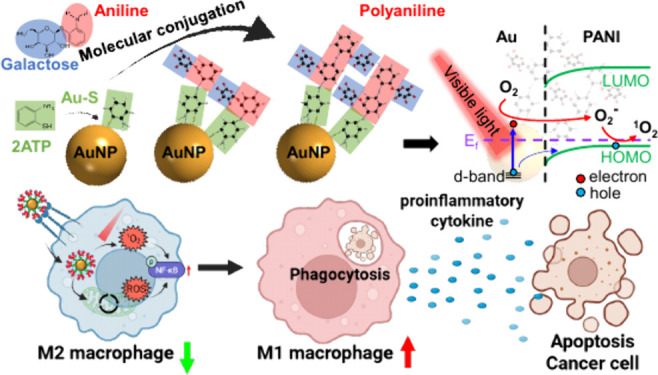

## Introduction

Light-responsive nanomaterials have emerged as powerful platforms in biomedical sensing, catalysis, and imaging, primarily due to their capability to modulate molecular and electronic structures when exposed to visible and near-infrared (NIR) light.^1–6^ In the deep-red to NIR range (650–1100 nm),^7,8^ the application of light in medicine has expanded for noninvasive stimulation and visualization within deeper tissues. Hybrid nanostructures that combine metals, semiconductors, and conductive polymers might not only integrate photobiostimulation with self-responsive imaging capability but also exhibit unique optoelectronic properties arising from interfacial molecular coupling.^2,5,9–12^ These multifunctional properties provide new avenues for the investigation of light-driven modulation and photoreactive behavior within living systems. Nevertheless, a substantial challenge persists in the design of biomedical materials endowed with optical activity that can facilitate biological recognition, imaging, tracking, and light-mediated cellular responses.

Nanoparticle (NP)-assisted phototherapy shows potential for effective delivery via the EPR effect, enhancing local photon absorption.^13^ Combining a targeting moiety with a self-trackable ability in nanostructures enables the active monitoring of reactive oxygen species (ROS)-induced bioreactions in the areas of interest.^2,8,14,15^ To harness the therapeutic immune-modulatory effects of nanoparticle-mediated phototherapy, a substantial amount of ROS is required to induce apoptosis and trigger the release of damage-associated molecular patterns (DAMPs)^16^ in cancer cells. However, due to the inherently hypoxic nature, achieving sufficient ROS generation in tumor is challenging in practice. In contrast, emerging approaches have explored the use of light-triggered ROS in immune cells to directly modulate immune response—particularly by reprogramming immunosuppressive M2-like macrophages toward the pro-inflammatory M1 phenotype.^15,17–20^ For example, organic hybrids^17–19^ have been demonstrated to directly assess M2-to-M1 reprogramming in macrophages by photodynamic therapy. Nevertheless, their delivery systems often lack selectivity and may cause additional phototoxicity to normal cells within the accumulation sites. A recent study investigated the use of up-conversion NPs (UCNPs) for converting NIR light into blue light to stimulate curcumin; however, the low excitation efficiency of UCNPs limits their therapeutic potential.^20^ The excitation of semiconductor NPs^15^ produces ROS for macrophage reprogramming, which also has potential but requires high power outputs, posing a risk of thermal damage to normal tissue.^21^ It is anticipated that if NPs modified with biosituatable molecules activate the immune response gene pathway, which can continuously promote M2-to-M1 polarization, regardless of whether it occurs before or after light exposure. To address these limitations, we propose enhancing the specific accumulation of photoactive and glycosylated NPs in macrophages by targeting the overexpressed galactose-binding receptors (MGL and CD206),^22^ as this enables the use of low-power LEDs or lower delivery doses.

In this study, we present a novel approach utilizing a simultaneous gold (Au) nanocatalyst and molecular conjugation strategy to facilitate a Au/2-aminothiophenol (2ATP)-assisted polymerization of a polyaniline (PANI)-based glycopolymer (PGlyco) via an one-pot reaction (Scheme 1). We hypothesize that the presence of the Au–S (2ATP) interfacial structure^21^ would enhance coupling with PGlyco, thereby promoting electron transfer between Au NPs and the conductive PANI structure.^22^ Electron paramagnetic resonance (EPR) results demonstrate that Au/2ATP@PGlyco NPs enhance the ^1^O_2_ concentration level, possibly through charge transfer involving Au/PANI to O_2_. Culturing M2-like macrophages with 20 ppm Au/2ATP@PGlyco NPs plus 660 nm led to 73% reprogramming of M1-like macrophages via photoinduced ROS generation (route 3–4 in Scheme 1) in M2-like macrophages, which is significantly higher than the 46% observed with Au@PGlyco NPs plus 660 nm and the 24% with Au/2ATP@PGlyco NPs alone. The latter is evolved by a galactose-mediated mitochondrial metabolic ROS generation (route 2 in Scheme 1) that complemented multivalent binding with glycopolymer moieties^22,23^ (route 1 in Scheme 1). High-sensitivity surface-enhanced Raman scattering (SERS) property^2,3^ of the PANI framework was utilized for single-cell imaging (at 671 nm), revealing this macrophage reprogramming in response to the ROS-mediated activation of inflammatory transcription factors NF-κB and STAT-1 (route 5). Furthermore, photoactivated M1-like macrophages perform phagocytosis and release inflammatory cytokines (TNF-α^24^ and IL-12 ^25^) that induce cancer cell apoptosis. This innovative optical nanohybrid, which enhances photo- and biostimulation with self-signaling, offers safer and more efficient treatment options for cancer patients, improving outcomes and understanding of photoimmune interactions with cancer cells during treatment.

**Scheme 1:**
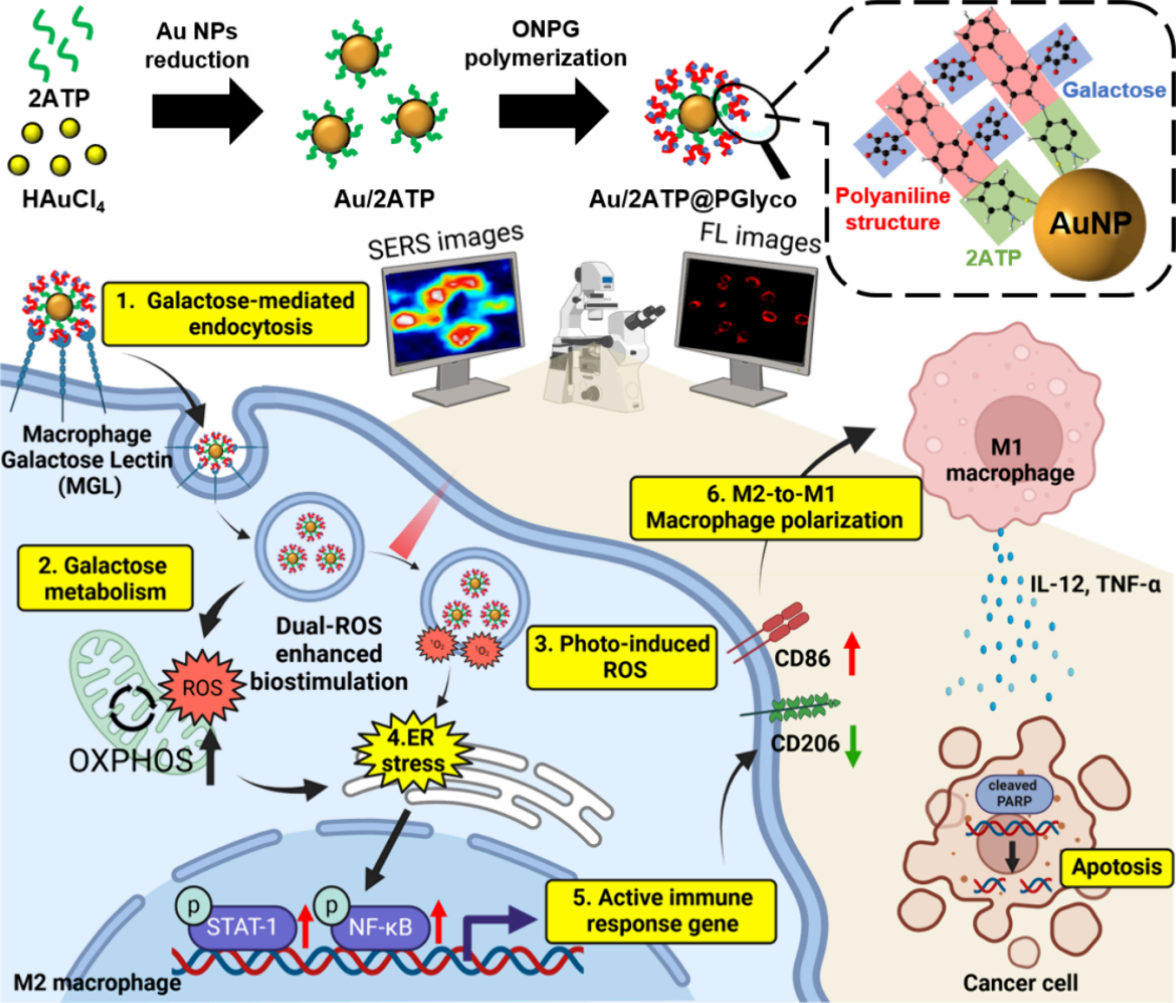
Illustrations of the Synthesis Process of Au/2ATP@PGlyco NPs from 2ATP-Immobilized Au NPs Followed by Meta Position Binding with a PANI-Based Glycopolymer, SERS Imaging Tracking Ability, Biostimulation/Photoactivated Macrophage Polarization, and the Proposed Mechanism for the Modulation of the Cellular Pathways of a Multivalent Binding to Galactose-Related Receptors (Route 1), Galactose-Mediated Mitochondrial Metabolism Activation (Route 2), Photoinduced ROS Generation (Route 3), Enhanced Activation of Endoplasmic Reticulum (ER) Stress (Route 4), and ROS-Induced NF-κB Pathway Activation (Route 5), Leading to M2-to-M1 Reprogramming (Route 6) and Inducing Cancer Apoptosis

## Results and Discussion

### Characterization of Au/2ATP@PGlyco Nanoparticles

As shown in Figure 1a, for a brief synthesis process, HAuCl_4_ was mixed with 2ATP molecules, followed by the addition of a NaBH_4_ reductant, resulting in metallic Au nanostructur. Next, ortho-nitrophenyl-β-galactopyranoside (ONPG) molecules were added for polymerization onto the Au NPs, forming Au/2ATP@PGlyco NPs through the reduction of ONPG by Au/NaBH_4_ reaction,^2^ followed by Au/Au^3+^-catalyzed polymerization of the amine-functional ONPG (Scheme S1). This process is detailed in our previous work on Au@PGlyco NPs.^2^ Figure 1b shows a TEM image of the Au/2ATP@PGlyco NPs, revealing a spherical structure with an approximate size of 44 ± 8.5 nm (Figure S1a, more than 100 particles). It exhibited a larger particle size than 18.5 ± 3.9 nm of Au@PGlyco NPs (Figure S2). Dynamic light scattering (DLS) analysis revealed that the hydrodynamic diameter of Au/2ATP@PGlyco NPs was 59.5 ± 9.3 nm, with a polydispersity index (PDI) of 0.023, indicating a good dispersity property in water (Figure S1b). The roughly 50 nm NPs may be appropriate for cellular endocytosis.^26^ Additionally, HR-TEM and EDS mapping images (Figure 1c) were used to examine the distribution of Au atoms combined with S elements from 2ATP to the Au NPs, as well as the N elements from PGlyco and 2ATP in the Au/2ATP@PGlyco NPs. The UV–vis spectra revealed that the as-synthesized Au/2ATP@PGlyco NPs exhibited an LSPR absorption peak at 537 nm (Figure 1d), which is higher than the 531 nm peak of the small-sized Au@PGlyco NPs.^2^ Compared to the Au/2ATP NPs at 534 nm and Au@PGlyco NPs at 531 nm, the LSPR was slightly red-shifted for Au/2ATP@PGlyco NPs. This can also be explained by an increase in the local refractive index (n) and dielectric constant (εd = n^2^)^27,28^ due to the codecoration of 2ATP@PGlyco on the surface of Au NPs. Polymers are reported to have higher refractive indices (1.45–1.6) than water at 1.33.^27^ Moreover, the LSPR absorption tail of the Au/2ATP@PGlyco NPs could generate SERS signals of PANI by interacting with 671 nm light. As shown in the SERS spectra (Figure 1e), the Au/2ATP and Au/2ATP@PGlyco NPs presented 2ATP-related signals at 901 cm^–1^, 940 cm^–1^, 1,112 cm^–1^, 1,422 cm^–1^, and 1466 cm^–1^, which were attributed to a possible in-plane orientation^29^ during Au/2ATP hybrid formation. The C–S bond^30^ at 1057 cm^–1^ was determined in relation to the thiol-terminal group of 2ATP to Au nanostructures,^31^ which evolved chemical^3^ and electromagnetic^2^ fields for SERS. In addition, the peaks at 1,159 cm^–1^, 1,235 cm^–1^, 1,314 cm^–1^ and 1,343 cm^–1^ were attributed to the increased polymerization in the PANI structure^2,9,32^ of the Au/2ATP@PGlyco NPs. Furthermore, the SERS spectrum (Figure S3a) of ten synthetic batches of as-prepared Au/2ATP@PGlyco NPs showed a low relative standard deviation (RSD) of 7.5% (Figure S3b), demonstrating highly reproducible SERS signals across independent syntheses.

**Figure 1:**
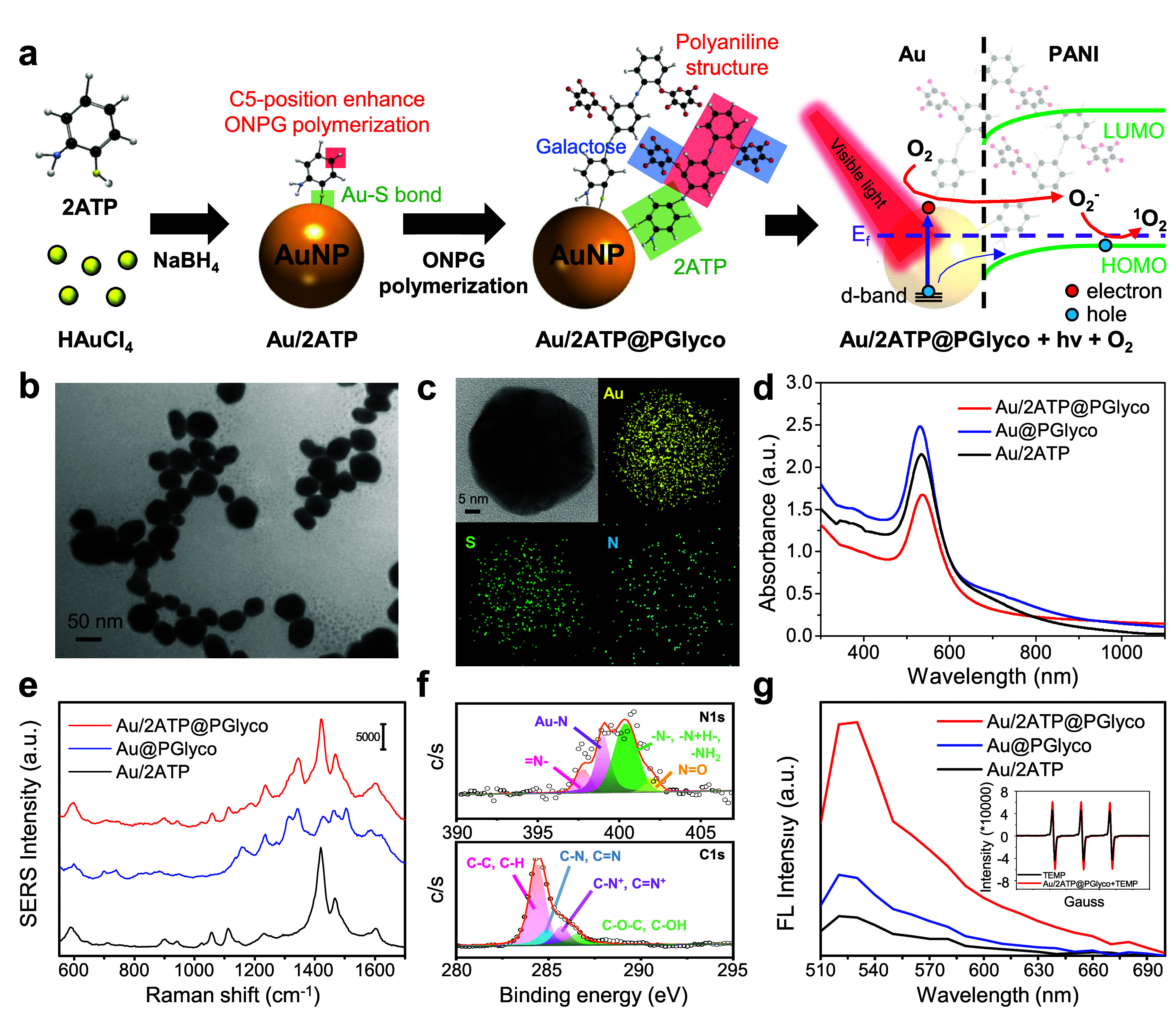
(a) Scheme for the synthesis of Au/2ATP@PGlyco NPs and the proposed generation mechanism from oxygen to superoxide to singlet oxygen by visible light excitation. (b) TEM image and (c) HR-TEM and EDS mapping images of the Au/2ATP@PGlyco NPs. (d) UV–visible spectra and (e) SERS spectra of Au/2ATP@PGlyco, Au@PGlyco, and Au/2ATP NPs. (f) XPS spectra for N 1s and C 1s of Au/2ATP@PGlyco NPs. (g) DCFH-DA assay for Au/2ATP@PGlyco, Au@PGlyco, and Au/2ATP NPs exposed to 660 nm LED (75 mW/cm^2^) for 15 min. The inset showed the EPR spectra for measuring ROS species of the Au/2ATP@PGlyco.

To further confirm the PANI structure around the surface of the Au/2ATP@PGlyco NPs, XPS spectra (Figure 1f) were used to analyze the molecular coating layer. The N 1s spectrum of the Au/2ATP@PGlyco NPs included 397.8 and 400.4 eV from the quinonoid imine (=N−), benzenoid amine (−N−) and protonated benzenoid amine (−N+·H−) structures,^33^ which were related to the PANI backbone.^2,22^ The C 1s spectrum were observed at 284.8 eV (C–N and C = N) and 285.4 eV (C–N^+^ and C = N^+^).^32^ The additional C 1s band at 286.5 eV of the Au/2ATP@PGlyco NPs originated from the galactose-based functional groups of the C–O–C and C–OH structures.^32^ As shown in Figure S4, the appearance of binding energies of 4f_7/2_ at 84.3 eV and 4f_5/2_ at 88.0 eV was identified as the formation of gold­(I)-thiolate complexes.^31,34^ The chemical states of Au, with binding energies of 83.7/87.4 eV (Au (0)) and 84.3/88.0 eV (Au (I)),)^34^ reflect the coexistence of Au atoms and gold­(I)-thiolate binding on the surface of Au/2ATP@PGlyco NPs. Furthermore, Benedict’s test revealed that the galactose content of the PGlyco of the Au/2ATP@PGlyco NPs was approximately 20% higher than the Au@PGlyco NPs (Figure S5a-c). This improvement could be attributed to the preinteraction of the 2ATP molecule with the Au NPs, which exposes the C5 position^2,9,35^ of the 2ATP and thereby promotes a favorable orientation for the reduced ONPG to conjugate at 2ATP. As the reaction with low 2ATP concentration resulted in low 2ATP and PGlyco-related SERS signals, it confirms that the conjugation of PGlyco relies on the pre-2ATP concentration at the surface of Au NPs (Figure S6a). Another control experiment, in which 2ATP was substituted with 4ATP (1,4 para-coupling benzene), led to the generation of weak intensity in the pattern feature of the PANI-based molecule (Figure S6b), attributed to the partial occupation of the free PGlyco on the Au/4ATP NPs (Figure S6b).

### Photoinduced ROS Generation by Au/2ATP@PGlyco Nanoparticles

DCFH-DA was used to analyze the ROS generation of the Au/2ATP@PGlyco NPs under the exposure of 660 nm LED. As shown in Figure 1g, the Au/2ATP@PGlyco NPs exhibited excellent ROS generation capability, showing a 6-fold increase compared to the Au/2ATP NPs without a PANI framework. Moreover, compared with the Au@PGlyco NPs (without the 2ATP molecular bridge), the ability of the Au/2ATP@PGlyco NPs to elicit ROS generation increased by approximately three folds. EPR spectra were used to analyze the ROS species with a 1:1:1 ratio of the three peaks, which is a typical observation of TEMPO signals (the inset in Figure 1g). The resulting generation of singlet oxygen (^1^O_2_) was improved in the presence of the Au–S interfacial structure in the Au/2ATP@PGlyco NPs. As shown in Figure S7, the EPR spectra displayed a similar trend to the DCFH-DA measurement (Figure 1g) of Au/2ATP@PGlyco > Au@PGlyco > Au/2ATP in ROS generation effectiveness. By measuring the decrease rate of absorbance at 440 nm with the singlet oxygen indicator (RNO test),^36^ the ^1^O_2_ generation capability from 660 nm light-excited Au/2ATP@PGlyco NPs showed improvement in an acidic environment (Figure S8).^37^ The acidification of PANI in the Au/2ATP@PGlyco NPs may have increased the charge transport property,^35^ which is related to the positive response to lysosomes (pH ∼ 4.5)^38^ within the cells.

According to previous research,^39^ PANI is a conductive polymer and acts as a p-type semiconductor organic material. Owing to the significant interactions between environmental molecules and oscillating electrons in gold NPs, recent studies^5,9–12^ have demonstrated that electron–hole pair separation occurs in photoexcited gold/semiconductor materials. The plasmonic hot holes take place from the d band of the Au NPs to the HOMO of the p-type semiconductor PANI backbone, as the system generates a photocurrent when the plasmon band is excited by visible–red light. The electrons in the Au counterpart might interact with the surrounding oxygen to form superoxide (Figure 1a).^4^ Temperini and co-workers verified the evolution of superoxide from the UV–visible photoexcitation of Au@PANI NPs.^9^ It was possible to subsequently oxidize superoxide induced by the hole in the PANI, leading to the formation of ^1^O_2_, similar to the reaction processes from the photoexcitation of the Au-semiconductor hybrid.^5,11,37^

### Bioactive and Light-Triggered Macrophage Reprogramming Efficacy by Au/2ATP@PGlyco Nanoparticles

To evaluate the effects of the biostimulation with the Au/2ATP@PGlyco NPs on macrophage reprogramming, we prepared M2-like macrophages from a murine cell line (RAW 264.7) for dark culture and light experiments (Figure 2a). No significant cytotoxicity was observed after 24 h of coculture with Au/2ATP, Au@PGlyco and Au/2ATP@PGlyco NPs, with or without light irradiation (Figure 2b). Flow cytometry analysis was used to verify macrophage reprogramming by determining the CD86 (M1 marker) expression. As shown in Figure 2c and S9, the M1 polarization efficiency of the Au/2ATP@PGlyco NPs (75%) was slightly higher than that of the Au@PGlyco NPs (70%) at 80 ppm, which is consistent with the increased PGlyco concentration in the Au/2ATP@PGlyco NPs (Figure S5). In contrast, the Au/2ATP NPs without the PGlyco molecular structure showed minimal reprogramming efficacy (Figure S9). Following hydrolysis of the galactose moiety with esterase and β-galactosidase before coculture with M2-like macrophages, the M2 to M1 reprogramming efficacy decreased (Figure S10). Reports indicate that the galactose moiety can induce oxidative phosphorylation (OXPHOS) in macrophages, generating adenosine triphosphate (ATP) for energy supply.^40^ This process may generate ROS, enhancing ER stress to active SYK kinase, leading to M2 to M1 macrophage polarization.^41,42^ Therefore, we used the ROS-ID Total ROS detection kit to measure the intracellular ROS levels in macrophages. Figure S11a showed that, compared with the Au/2ATP NPs, the Au@PGlyco and Au/2ATP@PGlyco NPs significantly increased the level of ROS in M2-like macrophages. To confirm polarization from enhanced OXPHOS by glycopolymers, we applied mitochondrial electron transport chain (ETC) inhibitors to block electron flow and ROS production (in route 2). Following treatment with the above inhibitors, the ROS levels (Figure S11b) and M2-to-M1 polarization (Figure S12) in the Au@PGlyco and Au/2ATP@PGlyco NP-treated M2-like macrophages significantly decreased. On the above basis, we proposed that galactose moieties^22,43,44^ on Au/2ATP@PGlyco NPs crucially target macrophages (route 1), which might modulate ROS/glycolysis-related metabolism^22^ on M2-to-M1 polarization (routes 4–5–6).

**Figure 2:**
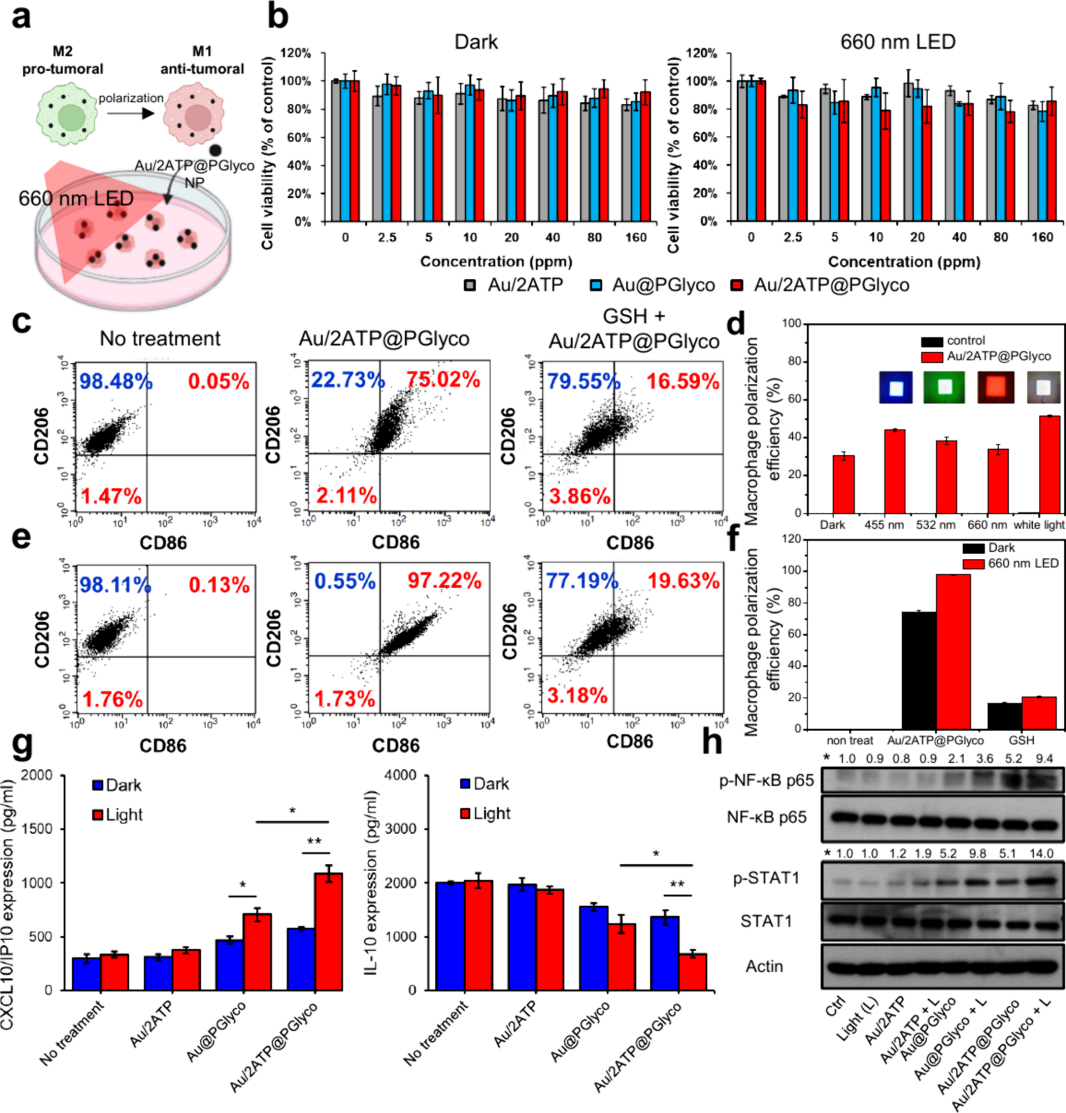
(a) Scheme of the Au/2ATP@PGlyco NP/light experimental process for the macrophage reprogramming test. (b) MTT assay for M2-like macrophages pretreated with Au/2ATP@PGlyco NPs for 24 h with or without 15 min of 660 nm LED irradiation (*n* = 4). (c) Flow cytometry evaluation (10,000 cells) of M2-to-M1 polarization induced by the Au/2ATP@PGlyco NPs in the dark condition. (d) Macrophage reprogramming efficiency of M2-like macrophages treated with Au/2ATP@PGlyco NPs under various light sources at 15 mW/cm^2^ (10,000 cells, *n* = 3). (e) Flow cytometry analysis (10,000 cells) of M2-to-M1 polarization by Au/2ATP@PGlyco NPs under 660 nm LED irradiation (75 mW/cm^2^) for 15 min. (f) Corresponding quantitative data from Panels (c) and (e) (10,000 cells, *n* = 3). (g) ELISA assay (*n* = 3) and (h) Western blot analysis of M2-like macrophages after 24 h of treatment with Au/2ATP@PGlyco NPs combined with or without light stimulation. The error bars represent SD (one-way ANOVA; *p < 0.05, **p < 0.01).

Notably, exposure of the Au/2ATP@PGlyco NPs to light (455 nm, 532 nm, 660 nm, white light) increased the M1-like macrophage reprogramming efficiency (Figure 2d, S13). RGB-integrated white light enabled the excitation of the NPs to produce ROS, facilitating the shift from M2 to M1. Deep red light at 660 nm offered better tissue penetration and lower phototoxicity,^45^ making it suitable for use in macrophage reprogramming experiments in the following proof-of-concept. When coculturing M2-like macrophages with low concentrations of 20 ppm Au/2ATP@PGlyco NPs and using 660 nm light, there was a 73% reprogramming of M1-like macrophages, attributed to the photoinduced generation of ROS in M2-like macrophages (Figure S14). This percentage is notably higher than the 46% reprogramming observed with Au@PGlyco NPs under similar light conditions (Figure S14), as well as the 24% achieved with Au/2ATP@PGlyco NPs alone (Figure S9). The M1 population presented at only 7.5% in the Au/2ATP+660 light group, suggesting the critical role of conductive PGlyco in the Au/2ATP interfacial structure (Figure S14).

Furthermore, we demonstrated a dose-dependent increase in M1-like phenotype in response to Au/2ATP@PGlyco NPs (Figure S9, S14). Enhanced photo fluxes over 0–30 min (Figure S15) and increased light doses (0–75 mW/cm^2^) (Figure S16) facilitated the M2-to-M1 polarization transition, suggesting the possible ROS-mediated macrophage polarization along routes 3–4–5–6 correlates with the M1 reprogramming efficiency.

Notably, the data in Figure 2e and S14 showed that 80 ppm Au/2ATP@PGlyco+660 nm light group offered effective remote macrophage reprogramming, achieving high reprogramming efficiency at 97%. This sample concentration could be a promising approach for reprogramming macrophages and warrants further investigation in anticancer research. The ROS levels in the M2-like macrophages cultured with the Au/2ATP@PGlyco NPs significantly increased after exposure to 660 nm light (Figure S11a and S17a). Adding glutathione, an antioxidant,^46^ inhibited M1 polarization (Figure 2c, 2e, and 2f) for Au/2ATP@PGlyco NPs by both dark and light treatments. Figures S17b and S18 showed that the mitochondrial inhibitors still kept the moderate photoinduced ROS generation and macrophage reprogramming under the exposure of 660 nm LED, highlighting the distinct reprogramming mechanism used by the route 3 pathway. Figures 2g and 2h reveal changes in CXCL10/IP10 secretion and activation of NF-κB and STAT-1 following 660 nm exposure, with a corresponding decrease in IL-10 due to M2 population suppression, being possibly triggered by the ER stress^41,42^ -mediated cascade reaction (route 4).

### Bio-SERS/FL Imaging for Investigating the Macrophage Reprogramming Process

Figure S19 presents a direct comparative analysis indicating that galactose moieties from the Au/2ATP@PGlyco NPs group accumulate more in M2-like macrophages than those from the Au/2ATP and Au@PGlyco NPs groups according to dark-field microscopy. Figure 3a shows ultramicrotome TEM images of the distribution of Au/2ATP@PGlyco NPs within lysosomes (red arrows) in the cytoplasm of M2-like macrophages. These Au/2ATP@PGlyco NPs might have an active and multivalent targeting effect on the binding of galactose-type lectin^23,47^ to M2-like macrophages, followed by receptor-mediated endocytosis (route 1 in Scheme 1). SERS-fluorescence microscopy (Figure 3b) of M2-like macrophages with Au/2ATP@PGlyco NPs was conducted to investigate single-cell discrimination. The background signal (black circle), recorded from the culture dish, did not show significant Raman peaks (Figure S20). By comparing the SERS intensities within living cells, the intensity at 1,343 cm^–^^1^ of the PANI vibrational mode of the PGlyco structure at the Au/2ATP@PGlyco NPs (Figure S20b) was chosen. The relatively strong SERS signal from the blue circle (Figure S20a-iii) indicated high levels of galactosylated NP accumulation within cells, allowing for precise particle-to-cell interactions by representing signal construction in x-y positioning. However, it revealed a significant decrease in SERS intensity with NaN_3_ pretreatment (Figure 3c),^6^ indicating that metabolic inhibition due to ATP depletion obstructs endocytosis. Adding galactose externally resulted in a ∼40% decrease in SERS intensity (Figure 3c), confirming that galactose-related receptors are essential for M2-like macrophage endocytosis of the Au/2ATP@PGlyco NPs. Figure 3d showed that there was no significant difference in the accumulation of NPs in M2-like macrophages between the Au/2ATP@PGlyco NP group and the Au/2ATP@PGlyco NP+660 nm light group. And each cell maintained a plume morphology without membrane injury according to bright field images. Notably, the Au/2ATP@PGlyco NPs+660 nm light group presented a relatively increased CD86 marker (red fluorescence for the M1-like phenotype) and a decrease in the CD206 marker (green fluorescence for the M2-like phenotype). In addition, the fluorescence generated from NF-κB phosphorylation^41,42^ was more pronounced in the Au/2ATP@PGlyco NPs+light treatment group than NPs alone group (Figure 3d). These findings suggest that Au/2ATP@PGlyco NPs combining light stimulation drive the evolution of antitumor immunogenic M1 reprogramming through multiple activations of PGlyco-elicited ROS stress (routes 2–4–5 in Scheme 1) and photoinduced singlet oxygen generation, followed by NF-κB-mediated pro-inflammatory signaling (routes 3–4–5 in Scheme 1).

**Figure 3:**
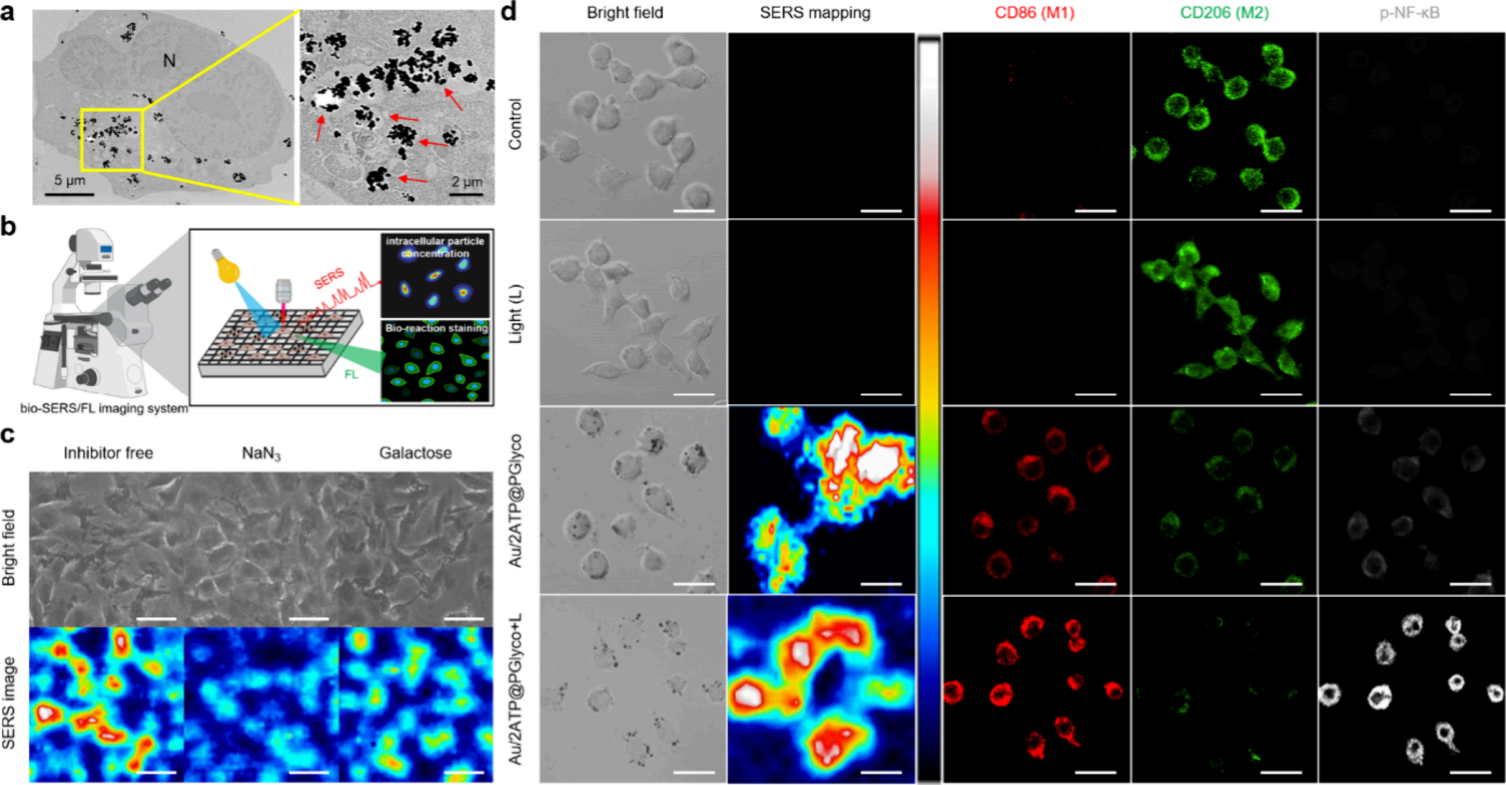
(a) TEM images of M2-like macrophages treated with Au/2ATP@PGlyco NPs for 24 h. (b) Scheme of bio-SERS/FL imaging for (c) analyzing the endocytosis process with SERS imaging and (d) demonstrating the expression of CD80-, CD206-, and p-NF-κB-related macrophages using integrated SERS/FL imaging. The scale bar is 20 μm.

### Multiple Fluorescence Imaging for Visualizing Macrophage-Cancer Cell Interactions in the 2D Coculture System

To evaluate the proinflammatory phenotype of M1-like macrophages to induce cancer cell death, we employed a 2D coculture system and examined the interaction between Au/2ATP@PGlyco NPs/light-treated M2-like macrophages and bladder cancer cells (MB49) using multiplex fluorescence staining (Figure 4a). In brief, macrophages were labeled with red fluorescence (Cell Tracking Dye Kit - Orange), cancer cells were labeled with blue fluorescence (Hoechst 33258), and dead cells were labeled with green fluorescence (Sytox Green). Multichannel fluorescence imaging (Figures 4b) revealed significant colocalization (Figure 4c) of green (dead cells) and blue (cancer cells) fluorescence, represented as cyan emission, indicating areas of cancer cell death for proof of concept. Approximately 77% of the cell death occurred after 12 h of coculture, as determined by the ratio of green to total blue and green fluorescent dots. In contrast, the untreated M2-like macrophage+MB49 and MB49 alone groups displayed only 7% and less than 2% cell death, respectively (Figure 4d and S21–22).

**Figure 4:**
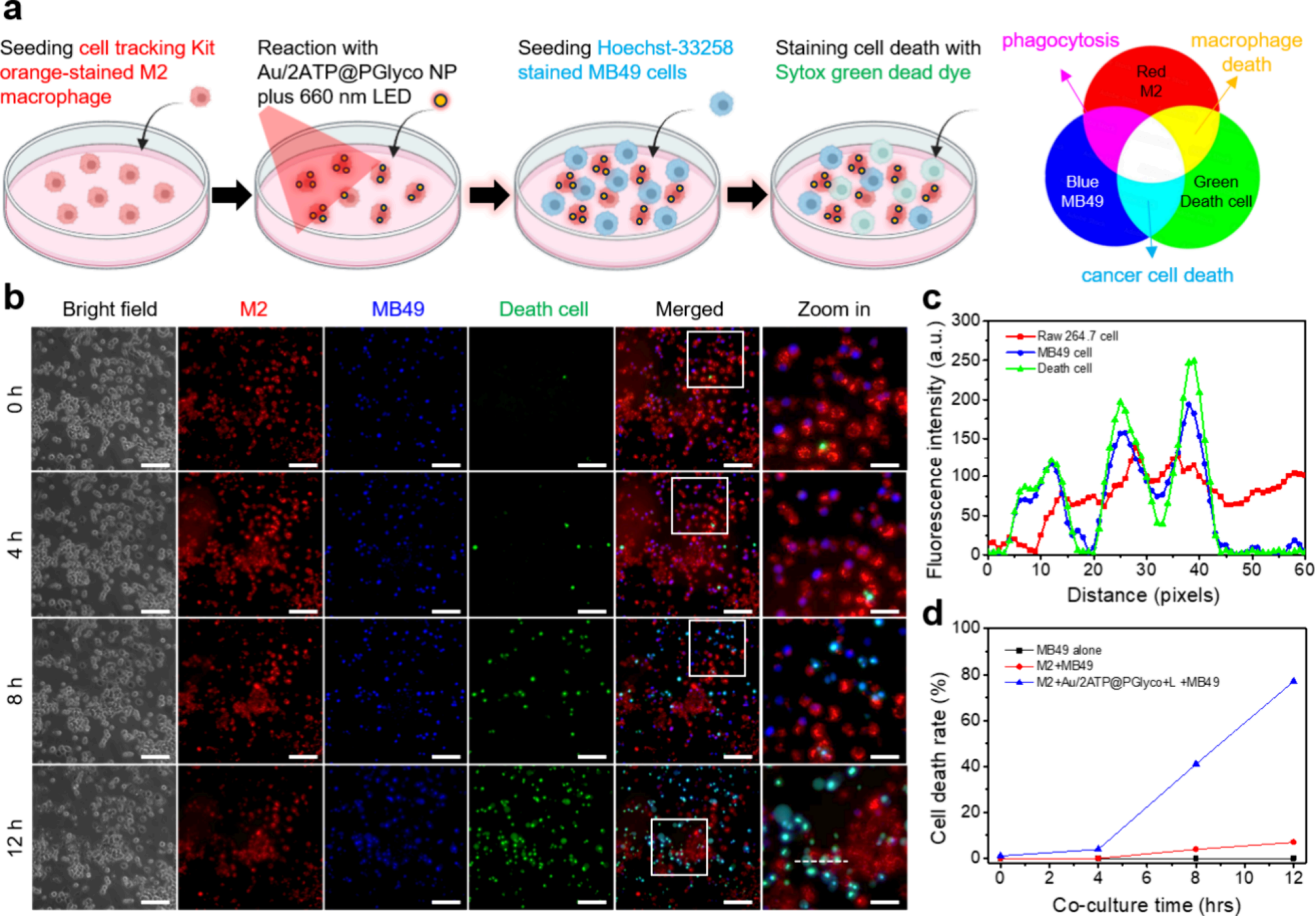
(a) Schematic representation of the 2D coculture process and multicolor staining for light–particle–cell interactions involving Au/2ATP@PGlyco NPs, M2-like macrophages, and MB49 cells. Results of (b) multiple fluorescence imaging (the scale is 100 μm), (c) fluorescence distribution along the dashed white line in (b), and (d) cell death rate in the coculture system after treatment with Au/2ATP@PGlyco NPs and light stimulation.

### Nanoparticle-Driven Macrophage Reprogramming Enhanced Proinflammatory Cytokine Release and Phagocytic Activity to Induce Cancer Cell Death

Moreover, we analyzed cytokine release and apoptosis-related proteins by collecting the medium and proteins from the coculture system. The Au/2ATP@PGlyco NP-treated macrophages significantly upregulated TNF-α (9.7-fold increase) and IL-12 (13.4-fold increase) secretion (Figure 5a), promoting cancer cell death via apoptosis, as confirmed by cleaved PARP^48^ expression (Figure 5b). As reported in the literature, increased levels of the proinflammatory cytokines TNF-α^24^ and IL-12,^25^ released from macrophages would enhance anticancer immunity. Additionally, M2-like macrophages treated with Au/2ATP@PGlyco NP combined with light also demonstrated greater phagocytic ability^49^ than those treated with Au/2ATP@PGlyco NP alone during a 12-h observation period did (Figure 5c). Notably, M2-like macrophages ± light groups exhibited negligible phagocytosis. Using scanning electron microscopy (SEM), we provided direct evidence of the interactions between regulated macrophages and cancer cells throughout the phagocytosis process (Figure 5d). Once the macrophages attached to MB49 cells at 6 h, their phagocytic activity visibly progressed to engulf cancer cells from 12 to 24 h. Although MB49 cancer cells (Figure S23) and macrophages (Figure 2b) were also exposed to light illumination, they exhibited less than 6% cell death at 24 h when treated with 80 ppm Au/2ATP@PGlyco NPs plus 660 nm light (75 mW/cm^2^). Hence, the death of these cancer cells is attributed primarily to the release of cytokines and the phagocytosis executed by M1-like macrophages. Besides identifying M2-like macrophages, we suggest that Au/2ATP@PGlyco NPs also selectively gather in bladder tumors via galactose-receptor binding,^50,51^ enhancing the antitumor immune response, which we will detail in our upcoming publication.

**Figure 5:**
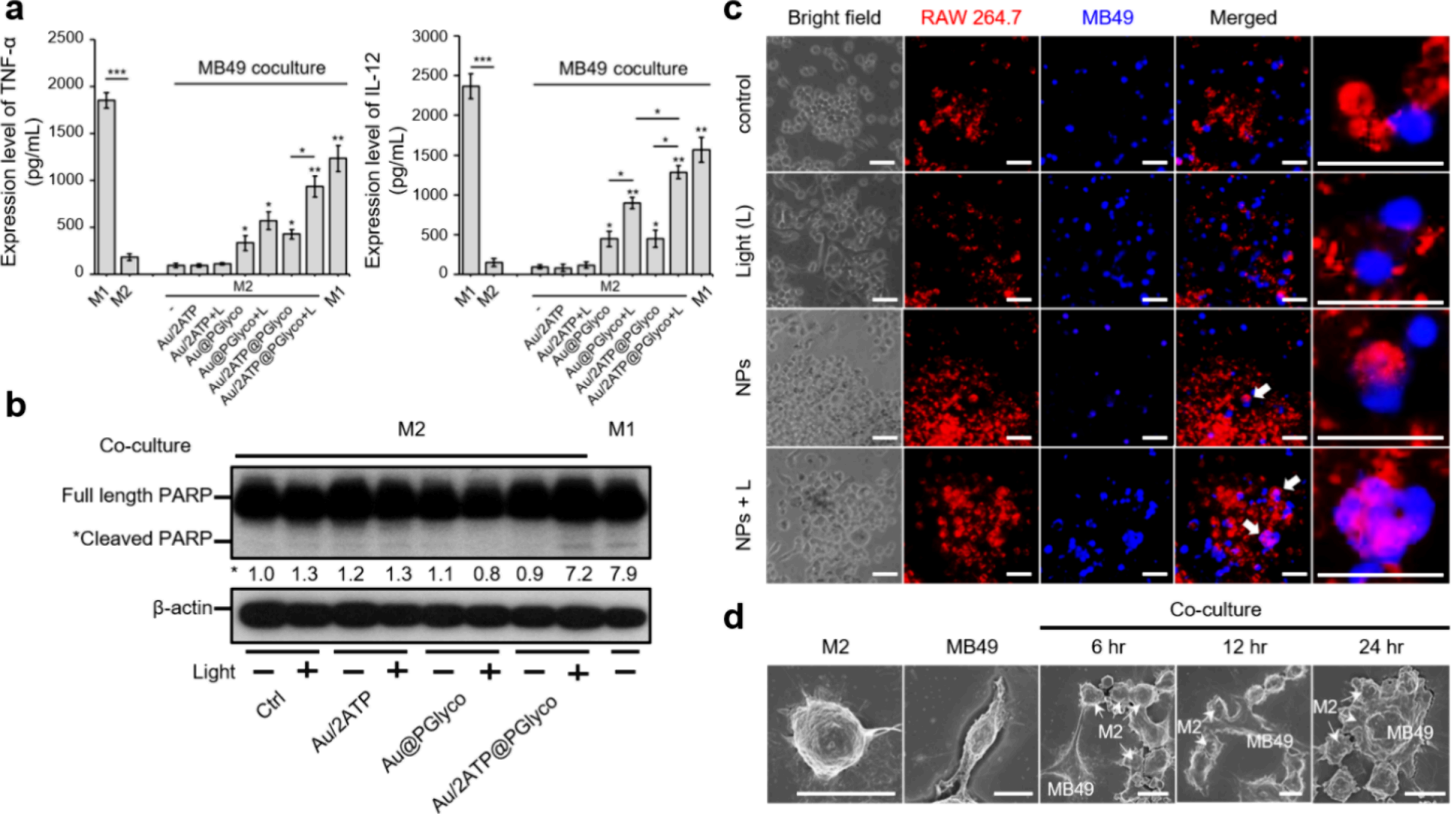
(a) ELISA analysis (*n* = 3) of TNF-α and IL-12 and (b) Western blot results collected from the medium and cells after 12 h of 2D coculture in microplates. Phagocytic effect analysis of M2-like macrophages/Au/2ATP@PGlyco NPs/660 nm light with MB49 cells in 2D culture experiments by (c) multiple fluorescence imaging at 12 h (the scale is 50 μm) and (d) SEM imaging at 6–24 h (the scale is 10 μm). The error bars represent SD (One-way ANOVA; *p < 0.05, **p < 0.01, ***p < 0.001).

Our recent mouse studies, which incorporate standalone galactosylated nanoparticles with immune checkpoint inhibitors, demonstrate considerable therapeutic potential against lung, pancreatic, and brain cancers.^22,42^ Importantly, these nanoparticles exhibit tumor accumulation and favorable biocompatibility. This study notably acts as a proof of concept, confirming that the combination of light and PGlyco-functionalized nanoplatform greatly enhances macrophage polarization toward the M1-like phenotype and bioactivity (Figure 2c, 2e, 5 and S14), thereby removing the necessity for extra animal models following the ‘3R’ principles in animal testing.

## Conclusions

In summary, the Au/2ATP nanocatalyst enhanced molecular conjugation with PANI-based polymers was developed to fabricate bio-optical Au/2ATP@PGlyco NP. This nanohybrid is capable of light-induced ^1^O_2_ generation and exhibit strong SERS signals based on the Au–S interfacial structure. These NPs could effectively target M2-like macrophages, inducing macrophage reprogramming through ROS activation driven by galactose metabolism and light-active treatments with low material and light dosages. The photostimulation of Au/2ATP@PGlyco NPs triggers the NF-κB signaling pathway, promoting their transition to M1-like macrophages and enhancing the release of cytokines and phagocytosis for anticancer purposes, monitored via SERS-fluorescence imaging. This presents a new pathway for exploring innovative light-responsive and modulated photoimmuno applications. It may enable clinical use of Au@2ATP@PGlyco NPs through intravesical instillation via cystoscope-guided fiber-optic illumination (660 nm) for effective photoimmunotherapy of bladder tumors and M2-like TAMs by the galactose-receptor reorganization.

## Experimental Methods

### Chemicals and Materials

Hydrogen tetrachloroaurate (III) trihydrate (HAuCl_4_·3H_2_O, 99%) and 3-(4,5-dimethylthiazol-2-yl)-2,5-diphenyltetrazolium bromide (MTT assay reagent) were purchased from Alfa Aesar. 2- Aminothiophenol (2ATP, 98%) was purchased from Thermos Scientific. Ortho-Nitrophenyl-β-galactopyranoside (ONPG, > 99%) was purchased from Biosynth Carbosynth. β-galactosidase (β-gal, from *E. coli*) was purchased from Sigma-Aldrich. Sodium tetrahydridoborate (NaBH_4_) was purchased from Aldrich. Phosphate-buffered saline (PBS, pH 7.0) was purchased from Thermo. Roswell Park Memorial Institute (RPMI) 1640 Medium, Fetal Bovine Serum (FBS), and Penicillin-Streptomycin were purchased from Gibco. Lipopolysaccharide (LPS), formaldehyde, and methanol were purchased from Merck. IFN-γ, IL-4, and IL-13 were purchased from Croyez Bioscience. Alexa Fluor 488 antimouse CD206 antibody, Alexa Fluor 594 antimouse CD86 antibody, and Alexa Fluor 647 antiphospho-NF-κB p65 antibody were purchased from BioLegend. Cell tracking Dye Kit-Orange-Cytopainter was purchased from Abcam. Bisbenzimide H 33258 (Hoechst 33258) was purchased from SIGMA. SYTOX Green Ready Flow Reagent was purchased from Invitrogen by Thermo Fisher Scientific. Enzyme-linked immunosorbent assay (ELISA) kits of CXCL10/IP10, IL-10, IL-12, and TNF-α were purchased from R&D Systems. Monoclonal antibodies of STAT-1, PARP, NF-κB, and p-NF-κB were purchased from Cell Signaling Technology.

### Preparation of the Au/2ATP, Au@PGlyco, and Au/2ATP@PGlyco NPs

Au/2ATP@PGlyco nanoparticles (NPs) were synthesized by first mixing 8 mL of deionized (D.I.) water with 2 mL of HAuCl_4_ (5 mM) under reflux heating at 80 °C for 10 min. Following this, 1 mL of 2-aminothiophenol (0.3 mM) was rapidly introduced while stirring vigorously for an additional 10 min. A 0.2 mL solution of NaBH_4_ (20 mM), acting as a reducing agent, was then added to the mixture and stirred for 1 min. Afterward, 1 mL of ONPG (75 mM) and HAuCl_4_ (5 mM) solution was injected into the mixture. After another minute of stirring, an additional 0.2 mL of NaBH_4_ (20 mM) was added to the stock solution and allowed to react for 5 min. To purify the Au/2ATP@PGlyco NPs, the solution was centrifuged at 6000 rpm for 10 min and washed three times with D. I. water. The purified samples were subsequently stored in D.I. water at 4 °C for use.

For the preparation of Au/2ATP NPs, a similar procedure was followed: a mixture of 8 mL of D.I. water and 2 mL of HAuCl_4_ (5 mM) was refluxed at 80 °C for 10 min. Then, 1 mL of 2-aminothiophenol (0.3 mM) was quickly added under vigorous stirring for 10 min. Finally, a 0.2 mL NaBH_4_ solution (20 mM) was added into the mixture for 1 min. To purify the Au/2ATP NPs, the solution was centrifuged at 6000 rpm for 10 min and washed three times with D. I. water. The purified samples were subsequently stored in D.I. water at 4 °C for use.

The Au@PGlyco NP was fabricated according to our previous work.^2^ A mixture of 8 mL of D.I. water and 2 mL of HAuCl_4_ (5 mM) was refluxed at 80 °C for 10 min. Then, 1 mL of ONPG solution (75 mM) was quickly added under vigorous stirring for 10 min. Finally, a 0.2 mL NaBH_4_ solution (20 mM) was added into the mixture for 5 min. To purify the Au@PGlyco NPs, the solution was centrifuged at 6000 rpm for 10 min and washed three times with D. I. water. The purified samples were subsequently stored in D.I. water at 4 °C for use.

### SERS Measurement of Solution Samples

Au/2ATP, Au@PGlyco, and Au/2ATP@PGlyco NPs were prepared in D.I. water with 100 ppm­[Au], and then 10 μL of the solution was dropped on the silicon substrate. The SERS spectrum was subsequently detected using a 671 nm laser (with a 20× objective, N.A. 0.45) with a power of 100 mW. The spectrum was employed with an integration time of 1 s with three accumulations.

### ROS Detections of the Au/2ATP, Au@PGlyco, and Au/2ATP@PGlyco NPs

The Au/2ATP, Au@PGlyco, and Au/2ATP@PGlyco NPs with a concentration of 100 ppm­[Au] were prepared according to atomic absorption measurements. These Au-based samples were mixed with DCFH-DA (20 uM), a wide variety of ROS detection indicators, and transferred to the LED board with different light excitation wavelengths for 30 min. Subsequently, the fluorescence spectrum was recorded by a microplate reader (Synergy H1, BioTek), and the fluorescence peak at 525 nm was measured to calculate the ROS generation.

For singlet oxygen detection, the samples (160 ppm) were mixed with RNO (0.1 mM) and imidazole (0.1 mM) as a singlet oxygen indicator. After a 1 min mixing time, the samples were exposed to a 660 nm LED (75 mW/cm^2^) for 30 min. The absorption peak intensity at 440 nm was measured every 10 min using a microplate reader (Synergy H1, BioTek), and the absorption change ratio was recorded to calculate the generation of singlet oxygen.

For ROS species identification, all samples (160 ppm) were mixed with TEMP (3 mM) and then exposed to a 660 nm LED board with a power of 75 mW/cm^2^ for 30 min. Then, the EPR spectrum was measured by an Electron Paramagnetic Resonance spectrometer.

### Cell Culture and Cell Viability Examination of Au/2ATP@PGlyco NPs

Murine macrophage Raw264.7 cells and mouse urothelial carcinoma MB49 cells were cultured in RPMI-1640 medium supplemented with 10% fetal bovine serum (FBS) and 1% penicillin/streptomycin (PS). All cell lines were maintained at 37 °C in a 5% CO_2_ incubator.

Living cells (5 × 10^3^) were seeded in 96-well plates and left to incubate overnight. Following treatment with varying concentrations of Au/2ATP, Au@PGlyco, and Au/2ATP@PGlyco NPs for 24 h, the cells were washed by centrifugation with phosphate-buffered saline (PBS) and then replaced with fresh medium. For the light stimulation group, cells were exposed to a 660 nm LED (75 mW/cm^2^) for 15 min. After an additional 24-h culture, a solution of 3-(4,5-dimethylthiazol-2-yl)-2,5-diphenyltetrazolium bromide (MTT reagent) at 1.2 mM was added to each well for 1 h. Subsequently, DMSO was added to dissolve the formazan crystals, and the absorbance was measured at 565 nm using a microplate reader (Synergy H1, BioTek).

### Macrophage Differentiation and Polarization

Raw264.7 cells (as M0-type) were seeded in a culture dish overnight. For M1-like induction, cells were treated with l00 ng/mL LPS and 20 ng/mL IFN-γ for 24 h. For M2-like induction, the cells were treated with 20 ng/mL IL-4 and 20 ng/mL IL-13 for 48 h.

### Bright- and Dark-field Microscopy of Au/2ATP@PGlyco NPs-Treated M2Macrophage

M2 macrophages (5 × 10^4^) were seeded in 18 × 18 mm^2^ coverslips overnight. Afterward, the M2 macrophages were treated with 80 ppm Au-based NPs for 6–24 h. After treatment, the cells were fixed with 4% paraformaldehyde (PFA) for 10 min and then centrifuged with PBS. The M2 macrophage was observed using a BX53 microscope (Olympus) equipped with both bright-field and dark-field capabilities for quantification of cell uptake.

### Flow Cytometry Analysis of Au/2ATP@PGlyco NPs-Treated M2Macrophage

M2 macrophages (1 × 10^5^) were seeded in 6 cm dishes and left to incubate overnight. Following this incubation, the M2 macrophages were treated with 80 ppm Au NPs for 24 h. The cells were then washed with PBS and refreshed with a new culture medium. For the light stimulation group, the particle-treated macrophages were exposed to a 660 nm LED (75 mW/cm^2^) for 15 min. After 24 h of culture, the macrophages were fixed using 4% formaldehyde for 15 min, permeabilized with ice-cold 100% methanol for 30 min, and subsequently washed with PBS. The cells were then stained with FITC-conjugated antimouse CD86 antibody and PE-conjugated antimouse CD206 antibody to differentiate the M1 and M2 phenotypes. Single-cell analysis was conducted using the BD FACSCalibur flow cytometer (BD Biosciences), and the data were processed with CellQuest Pro software.

To block mitochondrial electron transport chain function, M2 macrophages were pretreated with 2 μM Oligomycin (MedChemExpress), 1 μM Antimycin A (MedChemExpress), and 1 μM Rotenone (MedChemExpress) for 30 min before Au/2TP@PGlyco treatment.

### In Vitro ROS Detection via Flow Cytometry Analysis

M2 macrophages (1 × 10^5^) were seeded in 6 cm dishes and left to incubate overnight. Following this incubation, the M2 macrophages were treated with 80 ppm Au NPs for 24 h. The cells were then washed with PBS and refreshed with a new culture medium. For the light stimulation group, the particle-treated macrophages were exposed to a 660 nm LED (75 mW/cm^2^) for 15 min. Then, the cells were stained with ROS-ID Total ROS detection kit for 30 min. Subsequently, single-cell analysis was conducted using the BD FACSCalibur flow cytometer (BD Biosciences), and the data were processed with CellQuest Pro software.

To block mitochondrial electron transport chain function, M2 macrophages were pretreated with 2 μM Oligomycin (MedChemExpress), 1 μM Antimycin A (MedChemExpress), and 1 μM Rotenone (MedChemExpress) for 30 min before Au/2TP@PGlyco treatment.

### Living Cell SERS Detection and 2D Mapping Imaging

M2 macrophages (1 × 10^5^) were seeded in a 3.5 cm glass-bottom dish (Alpha Plus) overnight. After treatment with Au/2ATP@PGlyco NPs (80 ppm_[Au]_) for 24 h, the cells were washed with PBS, replaced with fresh medium, and subjected to SERS measurement using a BX53 microscope (Olympus) equipped with spectrometers (MRS-iHR 320). The Raman signals were acquired with a 671 nm laser at 3 mW (with a 40× objective, N.A. 0.75) for an integration time of 1 s. For SERS mapping imaging of cells, the SERS signals at 600 cm^–1^ were captured with 40 × 40 pixels over a mapping area of 200 × 200 μm^2^. To investigate the endocytosis pathway, the endocytosis inhibitor NaN3 (depleting ATP generation) or 10 mM galactose (MGL blocking) was pretreated for the M2 macrophages before treatment with Au/2ATP@PGlyco NPs.

### Bio-SERS/Fluorescence Microscopy for Macrophage Reprogramming Observation

M2 macrophages (1 × 10^4^) were seeded overnight in a 4-well chamber slide. After treatment with Au/2ATP@PGlyco NPs (80 ppm_[Au]_) for 24 h, the cells were washed with PBS and replaced with fresh medium. For the light stimulation group, the particle-treated macrophages were exposed to an additional 660 nm LED (75 mW/cm^2^) for 15 min. Following another 24 h of culture, macrophages were fixed with 4% paraformaldehyde for 10 min, permeabilized with 0.1% Triton X-100 for 20 min, and blocked with 1% BSA for 1 h. The cells were then stained with Alexa Fluor 488 antimouse CD206 antibody, Alexa Fluor 594 antimouse CD86 antibody, and Alexa Fluor 647 antiphospho-NF-κB p65 antibody for an additional hour. Subsequently, the stained macrophages were analyzed using a confocal fluorescence microscope (FV3000, Olympus) and SERS mapping with a 671 nm Raman system.

### Multiple Fluorescence Microscopy for Macrophage-Cancer Cell Interactions in the 2D Coculture System

M2 macrophages (1 × 10^5^) and MB49 cells (1 × 10^5^) were seeded overnight in 3.5 cm glass-bottom dishes. Then, the M2 macrophages were treated with Au/2ATP@PGlyco NPs (80 ppm_[Au]_) for 24 h, after which the cells were washed with PBS and replaced with fresh medium. For the light stimulation group, the particle-treated macrophages were exposed to an additional 660 nm LED (75 mW/cm^2^) for 15 min. For cell tracking, the M2 macrophages were labeled with the Cell Tracking Dye Kit-Orange-Cytopainter (red emission), and the MB49 cancer cells were labeled with Hoechst 33258 (blue emission) for 45 min. Subsequently, the Hoechst 33258-labeled MB49 cells were separated and seeded into the dish with the Cell Tracking Dye Kit-Orange-Cytopainter-labeled macrophages. Then, SYTOX Green Ready Flow Reagent (green emission) was added to track the dynamic cell death process of living cells. To observe the macrophage-cancer cell interactions, the coculture group was incubated for 12 h and recorded every 2 h.

### Scanning Electron Microscope (SEM) for the Phagocytosis Process

M2 macrophages (1 × 10^5^) and MB49 cells (1 × 10^5^) were seeded in 3.5 cm glass-bottom dishes overnight. Then, the M2 macrophages were treated with Au/2ATP@PGlyco NPs (80 ppm_[Au]_) for 24 h, after which the cells were washed with PBS and replaced with fresh medium. For the light stimulation group, the particle-treated macrophages were exposed to an additional 660 nm LED (75 mW/cm^2^) for 15 min. Subsequently, the MB49 cells were separated and seeded into the dish with macrophages for 6, 12, or 24 h. After incubation, the coculture cell group was fixed with 2.5% glutaraldehyde for 15 min and dehydrated using 1% hexamethyldisilazane for 15 min. Finally, the cells were dried in the hood overnight. The SEM images were analyzed using UHR-SEM (Hitachi SU8600).

### ELISA Analysis

The cell secretions of macrophages were collected from the culture medium and concentrated using a 3K centrifugal filter tube (Pall Corporation). Levels of CXCL10/IP10, IL-10, IL-12, and TNF-α were then detected using the DuoSet ELISA kit (R&D Systems) following the manufacturer’s instructions.

### Western Blot

Equal amounts of protein were loaded onto an 8% sodium dodecyl sulfate-polyacrylamide gel (SDS-PAGE) and subsequently transferred to polyvinylidene fluoride (PVDF) membranes (Millipore) over 2 h. The membranes were then blocked with 5% skim milk, rinsed with Tris-buffered saline containing Tween-20 (TBST), and incubated overnight at 4 °C with primary antibodies. The following day, secondary antibodies were applied for 1 h at room temperature, and the signals were detected using ECL Plus Western blotting detection reagents (Amersham). The densitometry function of ImageJ software (version 1.53r) was used to quantify the protein bands with gray values and shown as targeting protein/actin ratio or phosphorylated-protein/total-protein.

### Characterizations of As-Synthesized Nanoproducts

The nanoparticle morphology was obtained by transmission electron microscopy (TEM, Hitachi H-7500) at 80 keV. Energy-dispersive spectrometry (EDS) measurements for Au, C, O, and N element detection were performed by high-resolution transmission electron microscopy (HR-TEM, JEOL-2100F). The UV–visible absorption spectrum was recorded by an ultraviolet–visible spectrophotometer (UV–vis, JASCO V-730). The chemical states of Au, C, O, and N elements were identified by X-ray photoelectron spectroscopy (XPS, Thermo Fisher Scientific ESCALAB Xi^+^). The nanoparticle concentration was analyzed by atomic absorption spectroscopy (SensAA GBC, Australia). The ROS identification was obtained by an Electron Paramagnetic Resonance spectrometer (EPR, Bruker E580 CW/Pulse EPR). The SERS spectra were measured using a 671 nm Raman system (LiveStrong Optoelectronics, LS-PT 671) assembled with a power-adjustable 671 nm laser (100 mM, 0.1 nm full width at half-maximum) and a low noise spectrometer module (<13 cm^–1^ resolution). The hydrodynamic diameter of the NPs was measured with a dynamic light scattering spectrometer (Zetasizer Nano ZS90, Malvern, U.K.), and the PDI value was calculated by the equation below:

$$\mathrm{PDI}={\left(\frac{\sigma }{d}\right)}^{2}$$

where σ is the standard deviation of the particle size distribution and *d* is the mean particle size.

The flow assays were obtained by a BD FACSCalibur flow cytometer (BD Biosciences). The confocal fluorescence imaging was obtained by a confocal fluorescence microscope (FV3000, Olympus).

### Statistical Analysis

All experimental results were presented as the mean ± standard deviation (SD). Differences between groups were compared using one-way ANOVA, and a p-value below 0.05 was considered statistically significant.

## Supplementary Material



## Notes

Detailed experimental section, size distribution and DLS analysis (Figure S1), size distribution of Au@PGlyco (Figure S2), Repeatability test via SERS spectrum (Figure S3), XPS for Au 4f (Figure S4), Benedict’s test for galactose quantification (Figure S5), SERS spectrum for optimization of Au/2ATP@PGlyco NPs (Figure S6), EPR spectra (Figure S7), RNO test in acidic conditions (Figure S8) of Au/2ATP@PGlyco NPs. Macrophage reprogramming efficiency with different concentrations (Figure S9) and degraded by enzyme (Figure S10), ROS level analysis (Figure S11), macrophage reprogramming efficiency with mito-inhibitors treatment (Figure S12), macrophage reprogramming efficiency under different light source (Figure S13), macrophage reprogramming efficiency with different concentration under 660 nm light (Figure S14), macrophage reprogramming efficiency with different exposure times (Figure S15) and light power (Figure S16), ROS level (Figure S17) and macrophage reprogramming efficiency with mito-inhibitors treatment under 660 nm light (Figure S18), dark field images (Figure S19), Intracellular SERS imaging and corresponding SERS spectrum (Figure S20), multiple fluorescence imaging for anticancer efficacy (Figures S21 and22), cell viability of MB49 cancer cell (Figure S23) of the treatment of Au/2ATP@PGlyco NPs and related formats are provided in the Supporting Information (PDF)
