# Supplementary material for: Bioactive Light-Responsive Au Nanohybrids for Reactive Oxygen Species-Driven Macrophage Reprogramming
Source: ACS Appl Mater Interfaces. 2025 Sep 16;17(39):54453–65. doi: 10.1021/acsami.5c10407 (PMC12492335; doi:10.1021/acsami.5c10407)
Supplement: Supplementary file 1 [file am5c10407_si_001.pdf]

Supporting Information

Bioactive Light-Responsive Au Nanohybrids for ROS-Driven Macrophage Reprogramming

Ting-Yu Cheng (ORCID 0000-0003-2632-4073),[a,‡] Li-Chan Chang (ORCID 0000-0002-9953-5800),[b,‡] Li-Xing Yang (ORCID 0000-0002-3723-4391),[a] Sz-Syuan Wu,[a] Yu-Cheng Chin (ORCID 0000-0002-0302-7925),[a] Ya-Jyun Chen,[a] Zi-Chun Chia,[a] Wen-Pin Su (ORCID 0000-0003-0863-203X)[b,c,d,e,*] and Chih-Chia Huang (ORCID 0000-0002-9486-8665)[a,e,*]

[a]Department of Photonics, National Cheng Kung University, Tainan 701, Taiwan
[b]Institute of Clinical Medicine, College of Medicine, National Cheng Kung University, Tainan 704, Taiwan
[c]Departments of Oncology and Internal Medicine, National Cheng Kung University Hospital, College of Medicine, National Cheng Kung University, Tainan 704, Taiwan
[d]Clinical Medicine Research Center, National Cheng Kung University Hospital, College of Medicine, National Cheng Kung University, Tainan 704, Taiwan
[e]Center of Applied Nanomedicine, National Cheng Kung University, Tainan 70101, Taiwan

‡T.-Y.C., L.-C.C. are the first authors and contributed equally to this work.
*W.-P.S. and C.-C.H. are corresponding author contributed equally to this work.
*Corresponding authors e-mail: wpsu@mail.ncku.edu.tw (W.-P.S.), c2huang@mail.ncku.edu.tw (C.-C.H)

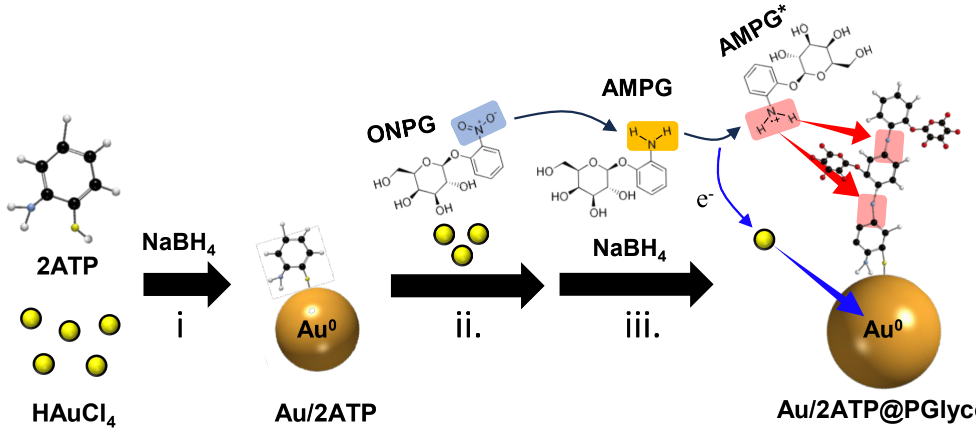

Scheme S1. Illustrations of the synthesis process and proposed reaction mechanism for Au/2ATP@PGlyco NPs. i) Initially, $HAuCl_4$ is reduced by $NaBH_4$ in the presence of 2-aminothiophenol (2ATP), forming Au/2ATP nanocatalysts. ii) Subsequently, the pre-mixed ortho-nitrophenyl-β-D-galactopyranoside (ONPG) and $HAuCl_4$ are introduced and iii) react with $NaBH_4$ in the Au/2ATP catalyst-including solution. According to previous literature,[1] the Au/2ATP nanocatalysts might facilitate hydrogenation of the nitro-group at ONPG to generate 2-Aminophenyl β-D-galactopyranoside (AMPG), followed by the oxidative coupling and polymerization reactions of the AMPG at Au/2ATP with the reduction of Au^{3+} species. The Au/2ATP NPs thus serve both as nucleation cores and catalytic sites for hydrogenation, and promote polymerization of AMPG to yield Au/2ATP@PGlyco NPs.

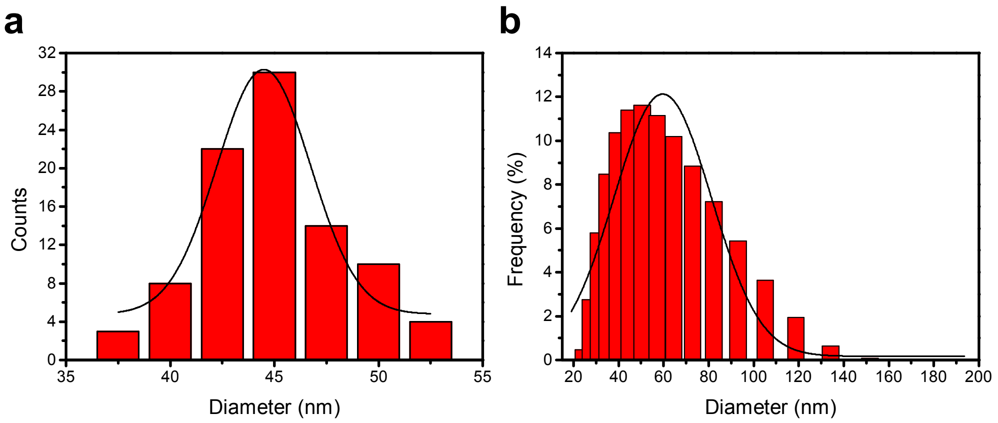

Figure S1. a) The size distribution of Au/2ATP@PGlyco NPs based on more than 100 particles from a TEM image in Fig. 1b of the main text. b) DLS analysis of Au/2ATP@PGlyco NPs.

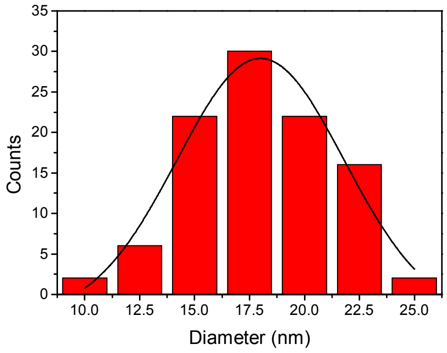

Figure S2. The size distribution of Au@PGlyco NPs based on more than 100 particles.

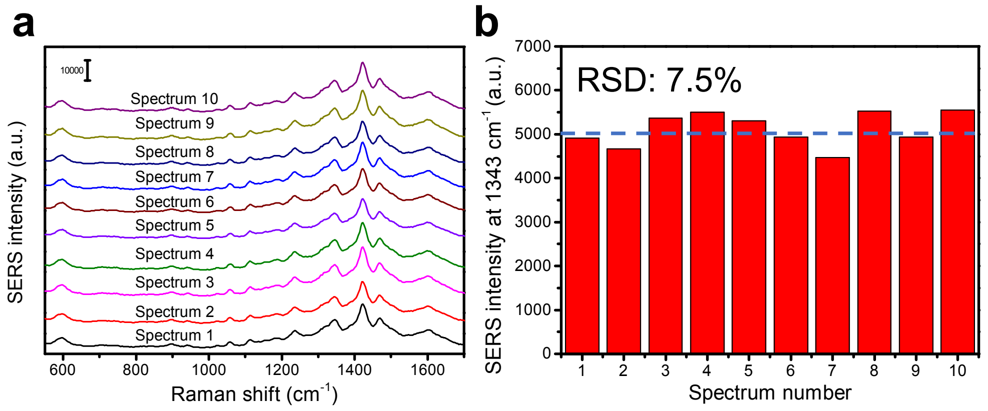

Figure S3. a) SERS spectra (at 671 nm) of 10 batches of Au/2ATP@PGlyco NPs with 50 $ppm_{[Au]}$. b) RSD value of SERS intensity at 1,343 cm^{-1} characteristic peak from 10 SERS spectra in (a).

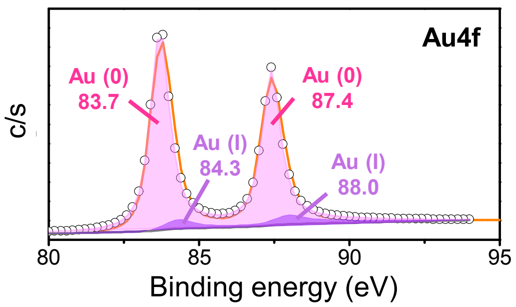

Figure S4. XPS spectrum for the Au4f peak of the Au/2ATP@PGlyco NPs.

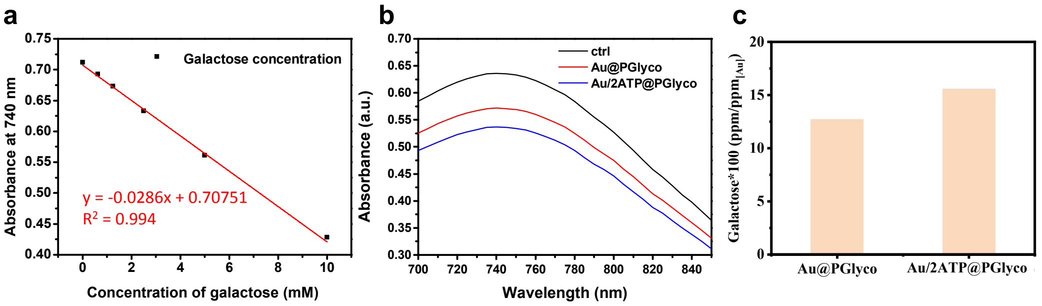

Figure S5. a) Calibration curve of 0–20 mM galactose with Benedict's test reaction via UV–visible records at OD740. b) UV–visible spectra of Benedict's reagent reacted with 5,000 $ppm_{[Au]}$ Au@PGlyco and Au/2ATP@PGlyco NPs. c) The galactose concentrations of Au@PGlyco and Au/2ATP@PGlyco NPs ($ppm/ppm_{[Au]}$) quantified with OD740 in the calibration curve of Benedict's test reaction from b).

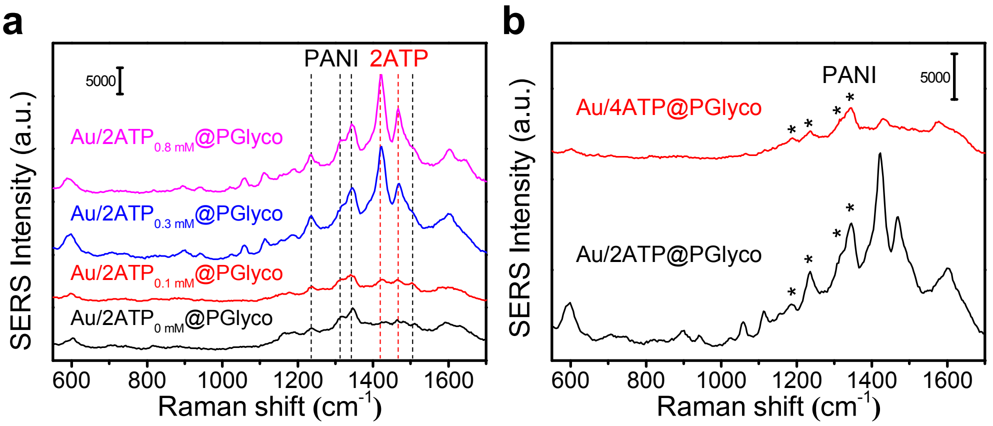

Figure S6. a) SERS spectrum of Au/2ATP@PGlyco NPs synthesized with different 2ATP concentrations. b) A comparison of the SERS spectra between Au/4ATP@PGlyco NPs and Au/2ATP@PGlyco NPs, both at the same concentration of Au.

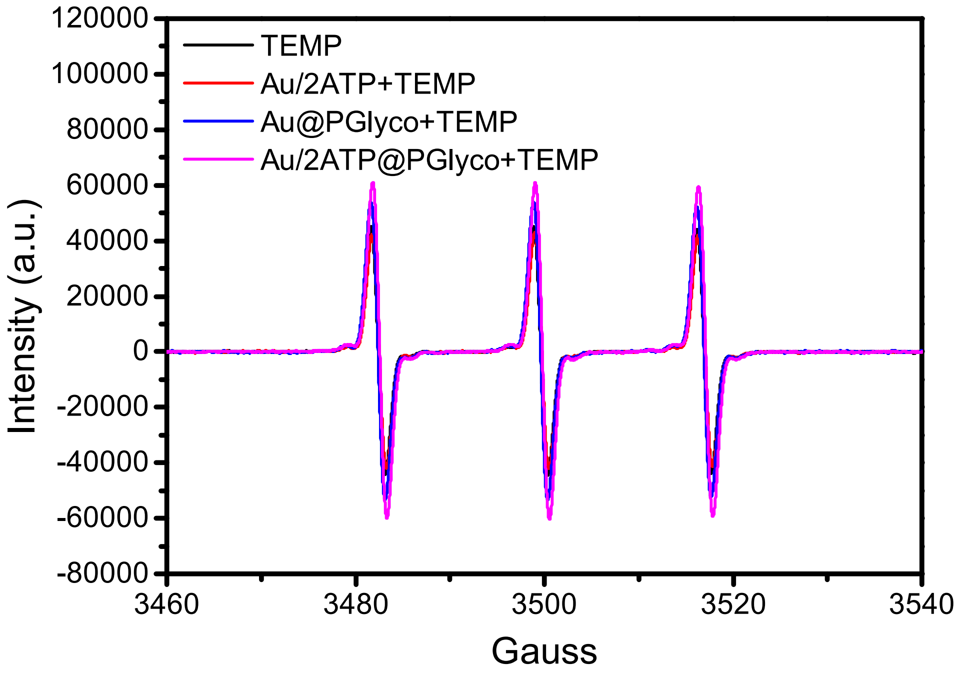

Figure S7. The EPR spectra for measuring ROS species of the 660 nm light-excited Au/2ATP@PGlyco, Au@PGlyco, and Au/2ATP NPs (160 $ppm_{[Au]}$).

Specifically, the reaction between singlet oxygen ($^1O_2$) and RNO/imidazole indicator to form an imidazole–$^1O_2$ intermediate, leading to the conversion of RNO into RNO_2. As shown in the following equation:

$$^1O_2 + \text{imidazole} \rightarrow [\text{imidazole} - {}^1O_2\ ]$$
$$[\text{imidazole} - {}^1O_2\ ] + \text{RNO} \rightarrow RNO_2 + \text{Products}$$

This reaction causes a decrease in absorbance of RNO at 440 nm in response to the generation of $^1O_2$ by Au/2ATP@PGlyco NPs plus 660 nm light. Therefore, a significant decrease in the RNO absorbance indicates a greater yield of singlet oxygen production.

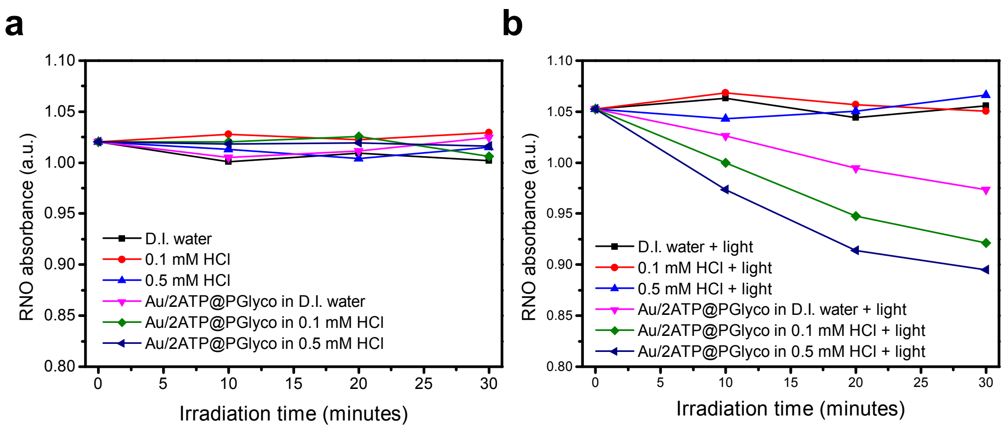

Figure S8. RNO test of particle-free solution (control group) and Au/2ATP@PGlyco NPs (160 $ppm_{[Au]}$) a) without and b) with a 660 nm LED (75 mW/cm^2) for 30 minutes in D.I. water and in 0.1 and 0.5 mM aqueous HCl solutions. The absorption peak intensity at 440 nm was measured every 10 minutes.

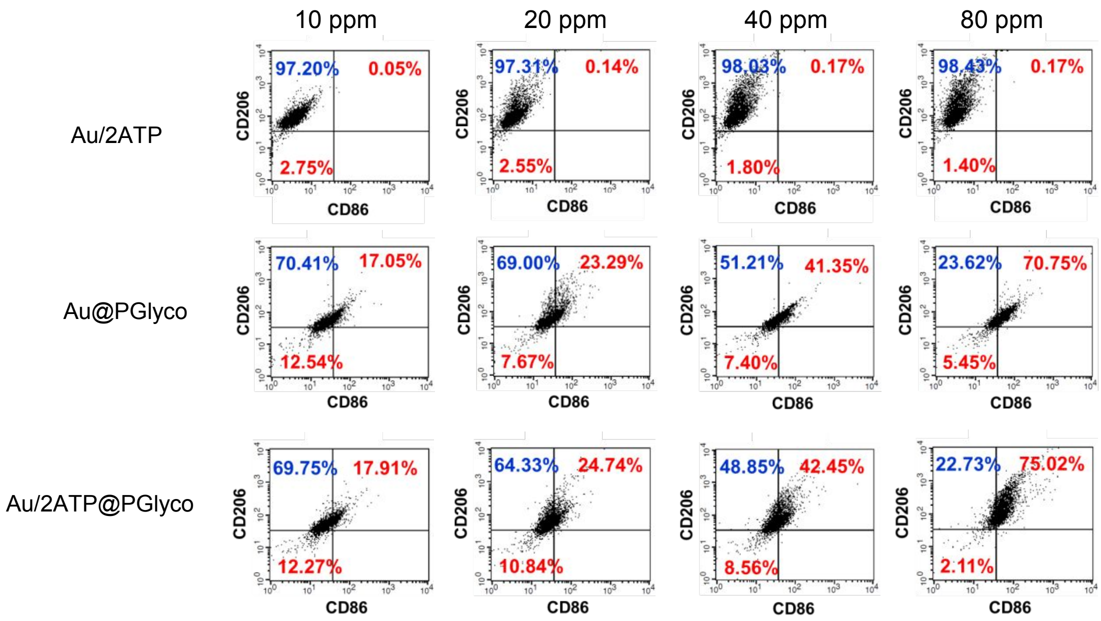

Figure S9. Flow cytometry analysis of M1 reprogramming efficiency by treating M2-like macrophages with Au/2ATP, Au@PGlyco, and Au/2ATP@PGlyco NPs for 24 hours at 10, 20, 40 and 80 $ppm_{[Au]}$ (10,000 cells counted).

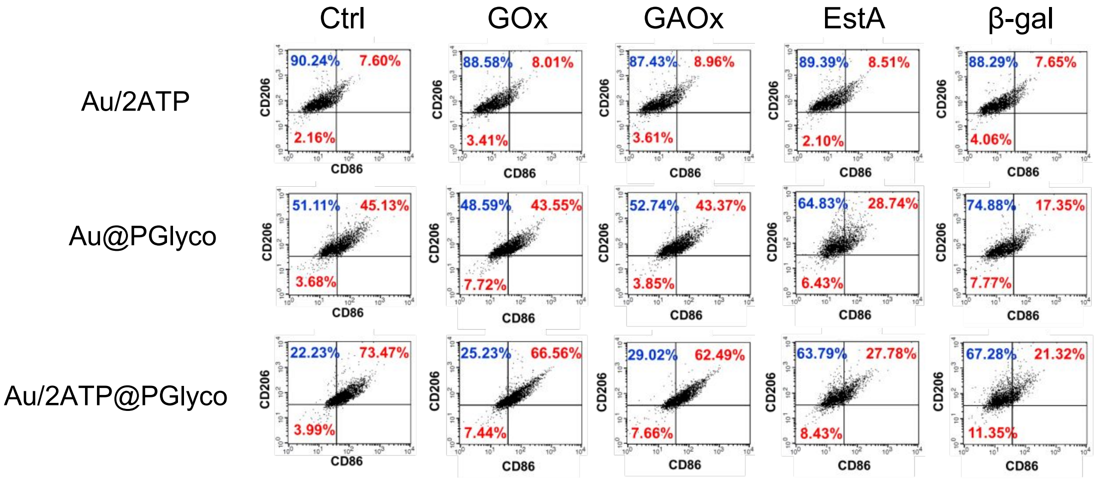

Figure S10. Flow cytometry analysis of M1 reprogramming efficiency by treating M2-like macrophages with Au/2ATP, Au@PGlyco, and Au/2ATP@PGlyco NPs (pre-treatments with GOx, GAOx, EstA, and β-gal for 24 hours) (10,000 cells counted).

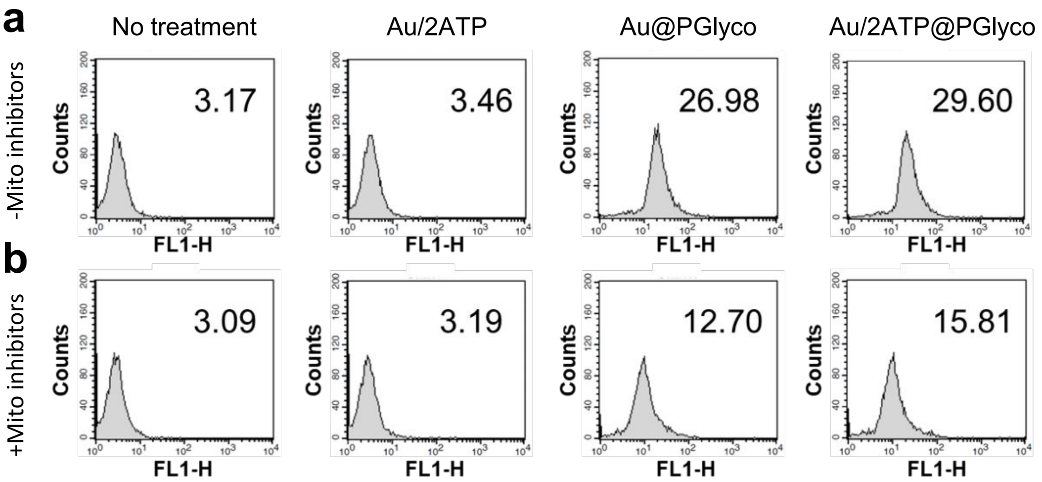

Figure S11. Flow cytometry evaluation of intracellular ROS levels in M2-like macrophages treated with Au/2ATP, Au@PGlyco, and Au/2ATP@PGlyco NPs under dark conditions a) without and b) after 30 minutes of pre-treatment with 2 μM oligomycin, 1 μM antimycin A and 1 μM rotenone (mitochondrial electron transport chain inhibitor) (10,000 cells counted).

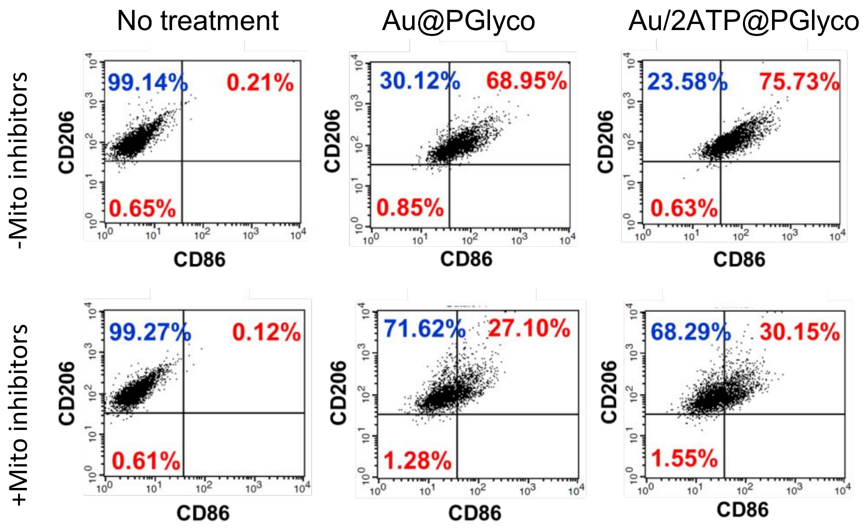

Figure S12. Flow cytometry evaluation of M2-to-M1 reprogramming by 80 ppm $_{[Au]}$ Au/2ATP@PGlyco NPs under dark conditions without and with 30 minutes of pretreatment with 2 μM oligomycin, 1 μM antimycin A and 1 μM rotenone (mitochondrial electron transport chain inhibitor) (10,000 cells counted).

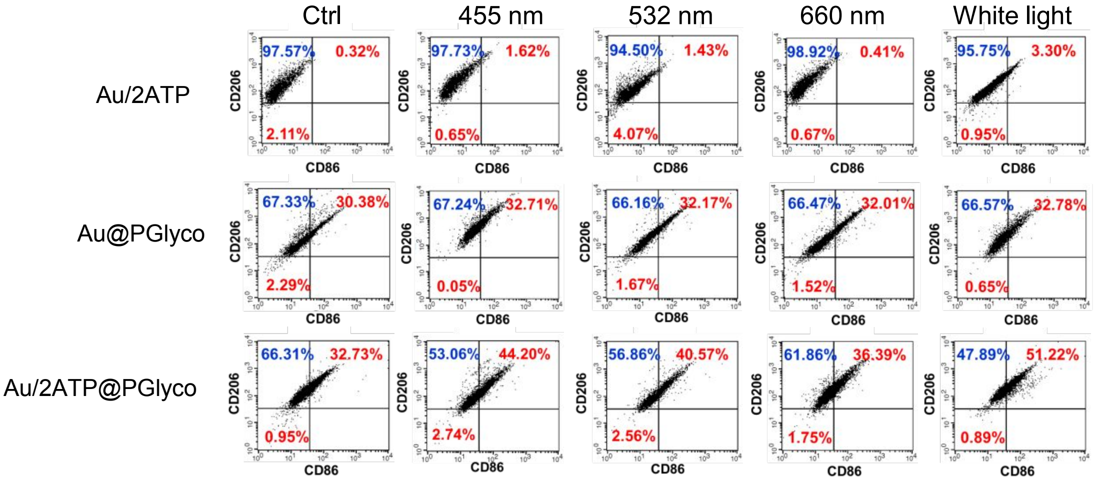

Figure S13. Flow cytometry analysis of M1 reprogramming efficiency by treating M2-like macrophages with Au/2ATP, Au@PGlyco, and Au/2ATP@PGlyco NPs for 24 hours at 20 $ppm_{[Au]}$ and different exposure light sources (455 nm, 532 nm, 660 nm, and white light LEDs with the light irradiation of 15 mW/cm^2) for 15 minutes (10,000 cells counted).

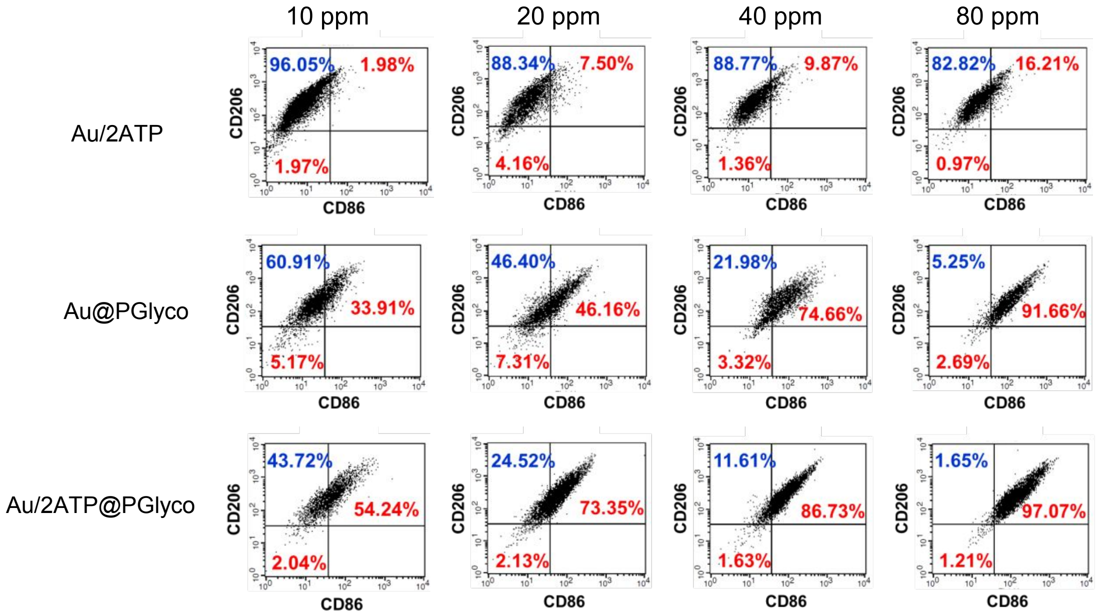

Figure S14. Flow cytometry analysis of M1 reprogramming efficiency by treating M2-like macrophages with Au/2ATP@PGlyco NPs for 24 hours at 10, 20, 40, 80 $ppm_{[Au]}$ and 660 nm LED (75 mW/cm^2) for 15 minutes (10,000 cells counted).

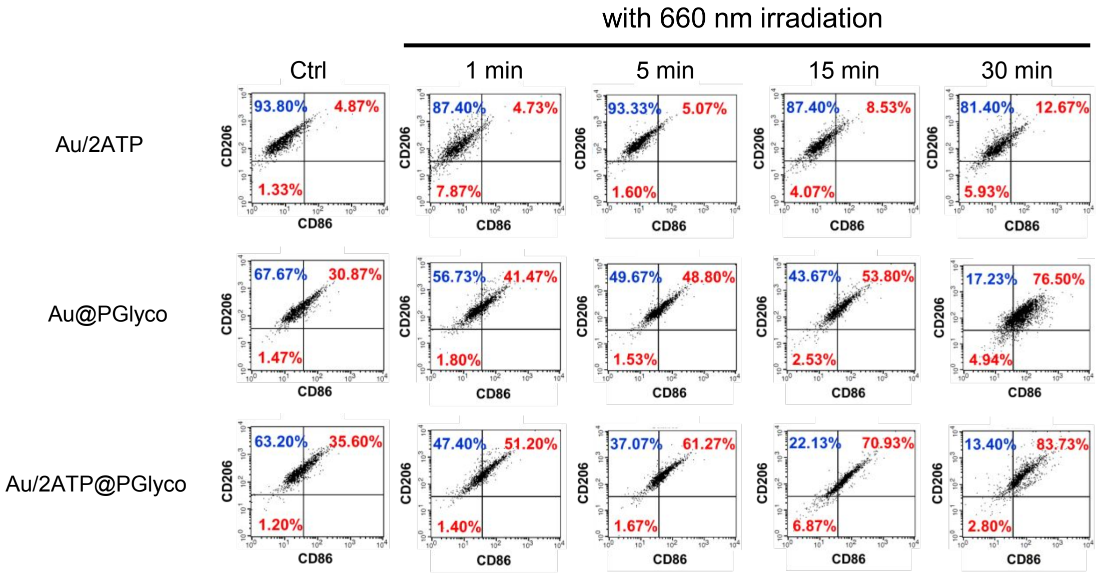

Figure S15. Flow cytometer evaluation of M2-to-M1 reprogramming by treating M2-like macrophage with 80 ppm$_{[Au]}$ Au/2ATP@PGlyco NPs for 24 hours with different exposure times (0, 1, 5, 15, and 30 minutes) of 660 nm LED (75 mW/cm^2) (10,000 cells counted).

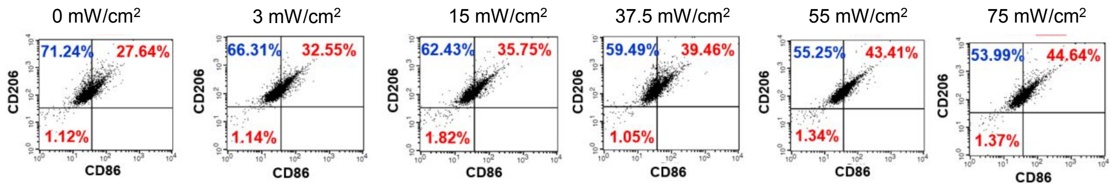

Figure S16. Flow cytometer evaluation of M2-to-M1 reprogramming by treating M2-like macrophage with 80 $ppm_{[Au]}$ Au/2ATP@PGlyco NPs for 24 hours with different exposure power (0, 3, 15, 37.5, 55, 75 mW/cm^2) of 660 nm LED for 15 minutes (10,000 cells counted).

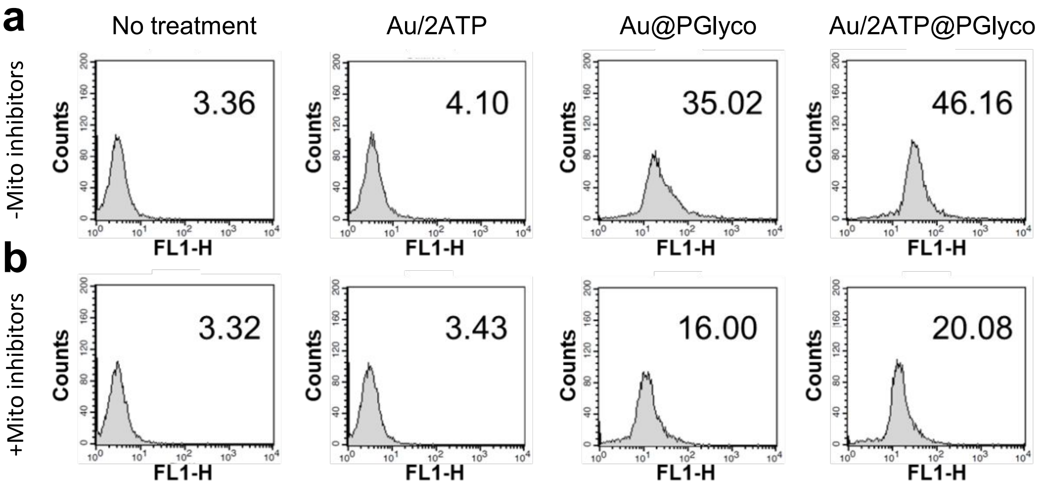

Figure S17. Flow cytometry evaluation of intracellular ROS levels in M2-like macrophages treated with Au/2ATP@PGlyco NPs under 660 nm LED irradiation (75 mW/cm^2) a) without and b) after 30 minutes of pretreatment with 2 μM oligomycin, 1 μM antimycin A and 1 μM rotenone (mitochondrial electron transport chain inhibitor) (10,000 cells counted).

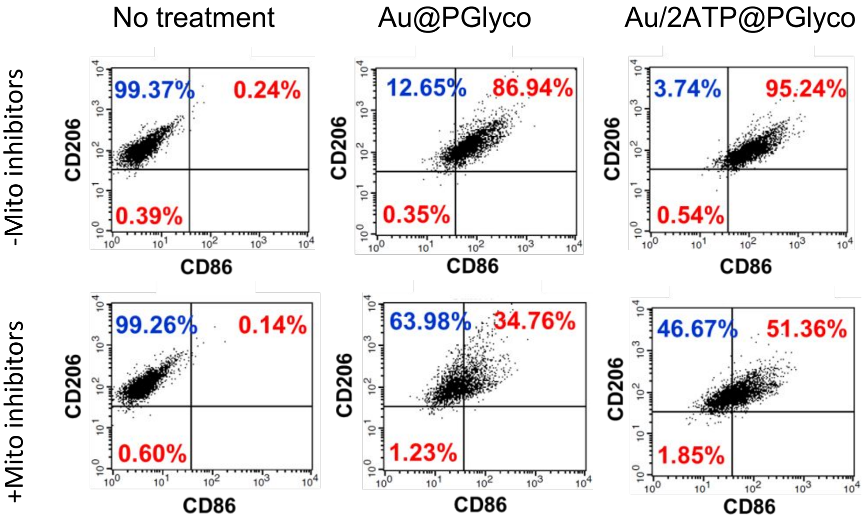

Figure S18. Flow cytometry evaluation of M2-to-M1 reprogramming by Au/2ATP@PGlyco NPs under 660 nm LED (75 mW/cm^2) irradiation without and with 30 minutes of pretreatment with 2 μM oligomycin, 1 μM antimycin A and 1 μM rotenone (mitochondrial electron transport chain inhibitor) (10,000 cells counted).

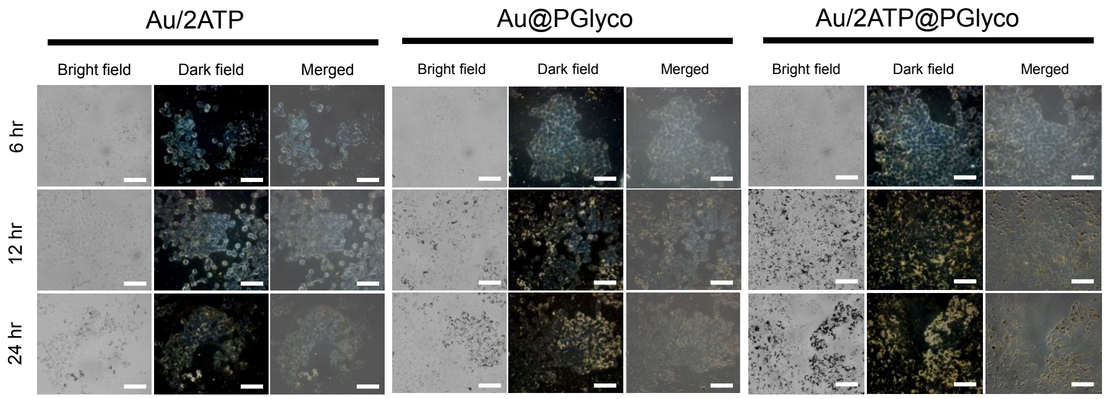

Figure S19. Dark field images of M2-like macrophages treated with 80 $ppm_{[Au]}$ of Au/2ATP, Au@PGlyco and Au/2ATP@PGlyco NPs for 6, 12, and 24 hours. The scale is 50 µm.

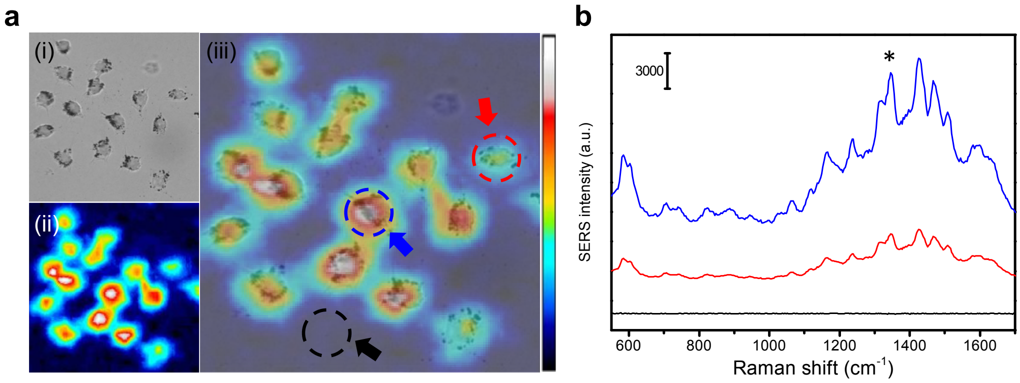

Figure S20. a-i) Bright field, a-ii) intracellular SERS imaging, a-iii) merged bright field-SERS imaging, and b) SERS spectra of M2 macrophage treated with 80 $ppm_{[Au]}$ of Au/2ATP@PGlyco NPs for 24 hrs. The line color represents the SERS signal corresponding to the same circle color area in (a-iii). The blue line indicates the high NPs-accumulation cell. The red line indicates the low NPs-accumulation cell. The black line indicates the background area.

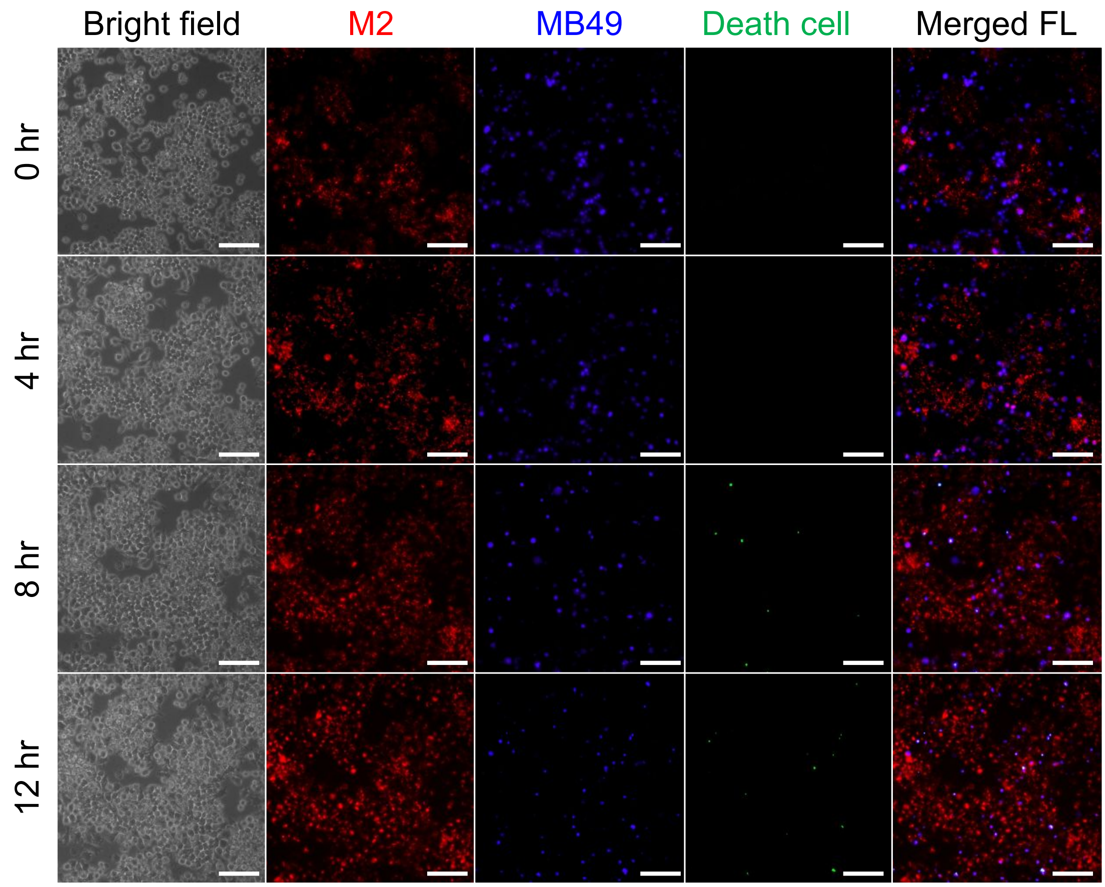

Figure S21. Multiple fluorescence images of M2-like macrophages alone (without particle and light treatment) cocultured with MB49 cancer cells for 0–12 hours. For fluorescence staining, the M2-like macrophages were labeled with a Cell Tracking Dye Kit-Orange-Cytopainter (red emission), and the MB49 cancer cells were labeled with Hoechst 33258 (blue emission). For dead cells, SYTOX^{TM} Green Ready Flow^{TM} Reagent (green emission) was used to track the dynamic cell death process of living cells. The scale is 100 μm.

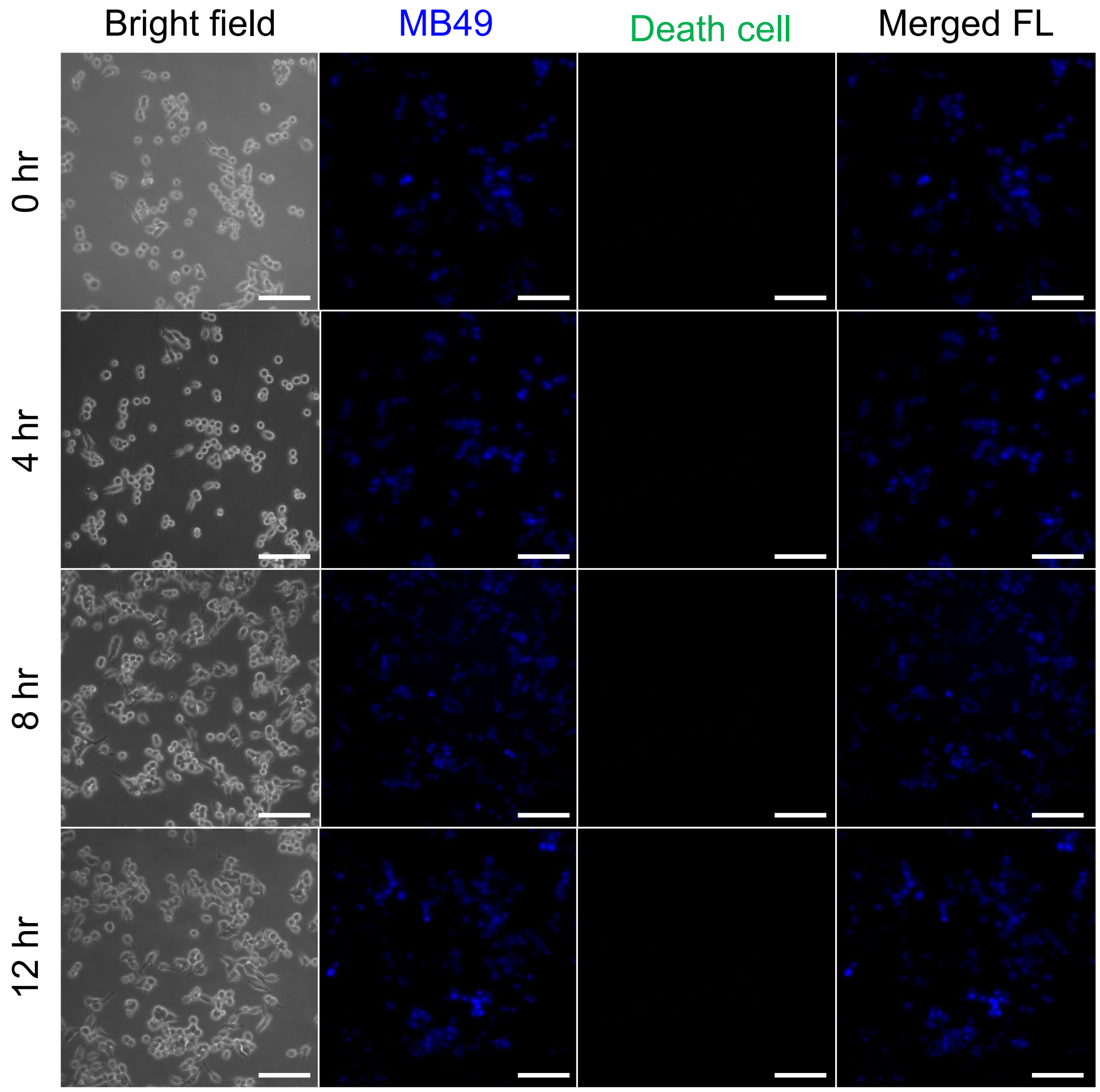

Figure S22. Multiple fluorescence images of MB49 cancer cells cultured alone for 0–12 hours. For fluorescence staining, the M2-like macrophages were labeled with a Cell Tracking Dye Kit-Orange-Cytopainter (red emission), and the MB49 cancer cells were labeled with Hoechst 33258 (blue emission). For dead cells, SYTOX™ Green ready Flow™ Reagent (green emission) was used to track the dynamic cell death process of living cells. The scale is 100 μm.

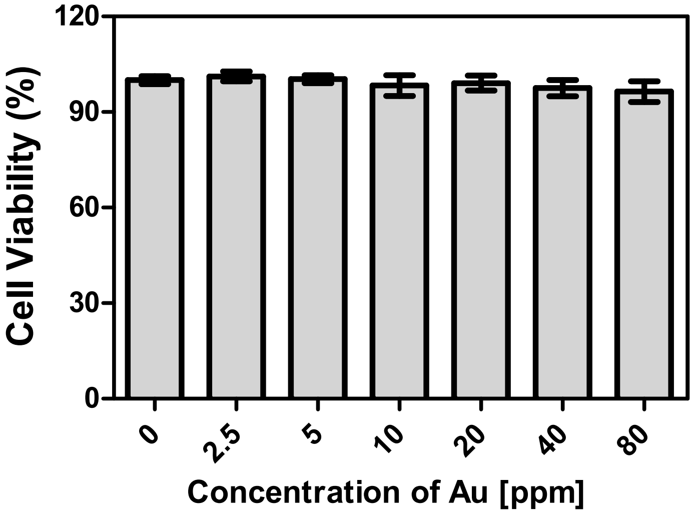

Figure S23. MTT assay for MB49 cancer cells that received Au/2ATP@PGlyco NPs with 10 minutes of 660 nm LED irradiation after 24 hours post-treatment (n=4).

Reference

(1) Chia, Z.-C.; Yang, L.-X.; Cheng, T.-Y.; Chen, Y.-J.; Cheng, H.-L.; Hsu, F.-T.; Wang, Y.-J.; Chen, Y.-Y.; Huang, T.-C.; Fang, Y.-S. In situ formation of Au-glycopolymer nanoparticles for surface-enhanced raman scattering-based biosensing and single-cell immunity. *ACS Applied Materials & Interfaces* **2021**, *13* (44), 52295-52307.
